# Gold(I) Complexes with a Quinazoline Carboxamide Alkynyl Ligand: Synthesis, Cytotoxicity, and Mechanistic Studies

**DOI:** 10.1002/ejic.202100120

**Published:** 2021-05-11

**Authors:** Leila Tabrizi, Won Seok Yang, Chetan Chintha, Liam Morrison, Afshin Samali, Joe W. Ramos, Andrea Erxleben

**Affiliations:** ^1^ School of Chemistry National University of Ireland Galway Galway Ireland; ^2^ University of Hawaii Cancer Center University of Hawaii at Manoa Honolulu USA; ^3^ Apoptosis Research Centre School of Natural Sciences National University of Ireland Galway Galway Ireland; ^4^ Earth and Ocean Sciences School of Natural Sciences and Ryan Institute National University of Ireland Galway Galway Ireland

**Keywords:** Alkynyl ligands, Cytotoxicity, Gold, Thioredoxin reductase, Translocator proteins

## Abstract

A series of gold(I) complexes with the general formula [Au(**L2**)(**L′**)] (**L2**=4‐phenyl‐*N*‐(prop‐2‐yn‐1‐yl)quinazoline‐2‐carboxamide, **L′**=PPh_3_ (triphenylphosphine), **1**; TPA (1,3,5‐triaza‐7‐phosphaadamantane), **2**, and Me_2_‐imy (1,3‐dimethylimidazol‐2‐ylidene), **3**) were synthesized and fully characterized by spectroscopic methods. The alkynyl ligand **L2** belongs to the quinazoline carboxamide class of ligands that are known to bind to the translocator protein (TSPO) at the outer mitochondrial membrane. **1** and **2** exert cytotoxic effects in bladder cancer cells with IC_50_ values in the low micromolar range. Further mechanistic analysis indicated that the two complexes both act by inducing reactive oxygen species and caspase‐mediated apoptosis. The complexes inhibit thioredoxin reductase, an established target of anticancer gold(I) complexes. Docking studies confirmed that after ligand exchange the free ligand **L2** can interact with the TSPO binding site.

## Graphical abstract

Alkynyl gold(I) complexes with phosphane and NHC co‐ligands have been prepared that are potent inhibitors of thioredoxin reductase. The complexes containing phosphane co‐ligands are also potent cytotoxins that reduce cell viability by inducing reactive oxygen species and caspase‐dependent apoptotic cell death.

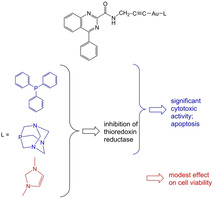

## Introduction

Gold compounds have a long history in medicine. At the end of the 19^th^ century Robert Koch described the bacteriostatic activity of K[Au(CN)_2_]^[1]^ and in the 1930s the effect of Au compounds on rheumatoid arthritis was discovered.^[2]^ The most prominent gold‐based rheumatoid arthritis drug, auranofin ((1‐thio‐β‐D‐glucopyranosato)(triethylphosphine)gold 2,3,4,6‐tetraacetate), which was approved by the FDA in 1985, was shown to also possess significant *in vivo* activity against P388 murine leukemia.[^3^, ^4^] The discovery of the antiproliferative effects of auranofin has led to an increased interest in Au complexes as an alternative to the clinically used Pt cancer drugs which have several drawbacks such as toxicity and inherent and acquired resistance.^[5]^

In contrast to Pt drugs that interact with DNA, Au compounds appear to target proteins and enzymes where Au coordinates to sulfur and selenium donor atoms. Although the mode of action is probably complex and multifactorial, there are increasing indications that the selenoenzyme thioredoxin reductase (TrxR) is the primary biological target of anticancer Au compounds.[^6^, ^7^, ^8^] Different classes of Au complexes have been evaluated to date; (i) neutral linear auranofin‐type phosphane complexes, (ii) tetrahedral cationic Au(I) phosphane complexes, usually containing chelating bis(phosphane) ligands, (iii) linear Au(I) *N*‐heterocyclic carbene (NHC) complexes and (iv) square‐planar Au(III) complexes.[^9^, ^10^, ^11^, ^12^, ^13^, ^14^, ^15^, ^16^, ^17^, ^18^] Recently a few [Au(I)(alkynyl) (phosphane)] complexes have been reported that showed promising *in vitro* and *in vivo* activities, although their number has remained small compared to other gold complexes.[^19^, ^20^, ^21^, ^22^, ^23^]

**Figure 1:**
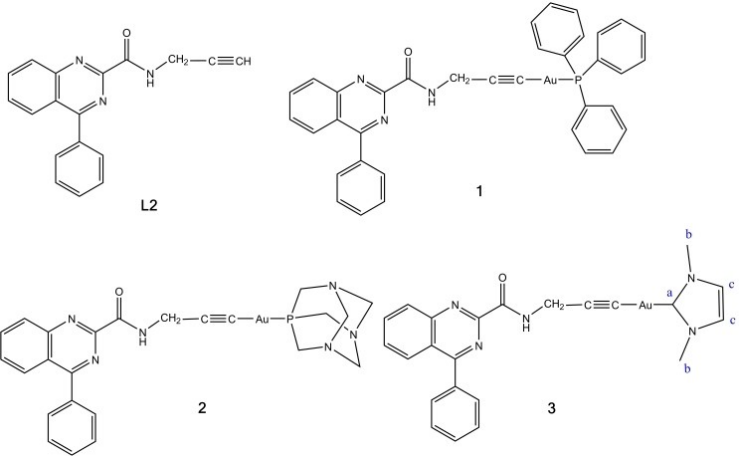
Structures of **L2** and complexes **1**–**3**.

Here we report alkynyl phosphane and alkynyl NHC Au(I) complexes where the alkynyl ligand is a derivative of the quinazoline carboxamide class of ligands that bind to the translocator protein 18 kDa (TSPO). TSPO is a relatively small transmembrane protein on the outer mitochondrial membrane. It plays a fundamental role in mitochondrial biochemistry and quality control, the regulation of the energy metabolism, the transport of heme precursors into the mitochondria, steroidogenesis, immunomodulation and cell proliferation.^[24]^ TSPO is overexpressed in many tumor types and the level of overexpression correlates with tumor malignancy and cancer progression.[^25^, ^26^] Compounds that interact with TSPO can chemosensitize solid tumors and are investigated as diagnostic and therapeutic agents[^27^, ^28^, ^29^, ^30^, ^31^] as well as carriers for selective drug delivery.[^32^, ^33^] TSPO binders have also been used as bioactive ligands in metallodrugs. Natile and coworkers modified TSPO binding agents with a metal binding site for Pt(II) and Re(I)[^34^, ^35^, ^36^, ^37^, ^38^] and Margiotta and coworkers reported a dual‐action Pt(IV) pro‐drug of oxaliplatin containing a derivative of the TSPO ligand alpidem.^[39]^ A TSPO‐targeting Cu imidazopyridine complex with promising *in vivo* activity has recently been published by us.^[40]^ Inspired by the work of Castellano *et al*. who developed TSPO ligands based on the 4‐phenylquinazoline‐2‐carboxamide scaffold^[41]^ we have also studied the biological activities of Pt(IV) complexes of 3‐(4‐phenylquinazoline‐2‐carboxamido) propanoate.^[42]^ We have now synthesized the alkynyl‐functionalized ligand **L2** and its Au(I) complexes [Au(**L2**)(PPh_3_)] (**1**), [Au(**L2**)(TPA)] (**2**) and [Au(**L2**)(Me_2_‐imy)] (**3**) (**L2**=4‐phenyl‐N‐(prop‐2‐yn‐1‐yl)quinazoline‐2‐carboxamide, TPA=1,3,5‐triaza‐7‐phosphaadamantane and Me_2_‐imy=1,3‐dimethyl‐imidazol‐2‐ylidene, Figure 1). We have evaluated the cytotoxic activities of the Au(I) complexes and investigated their mode of action in bladder cancer cell lines. Bladder cancers are one set of cancers in which Pt cancer drugs have been used. Response rates to cisplatin‐based chemotherapy of bladder cancers are around 50 %.^[43]^ Thus, this cancer with known metallodrug response may be one that could benefit from a new class of metal compounds that may have potentially better response rates and potentially better tolerance with fewer or less severe side effects. To the best of our knowledge this is the first design of anticancer gold complexes with a TSPO binding ligand.

## Results and Discussion

### Synthesis and Characterization

The ligand 4‐phenylquinazoline‐2‐carboxylic acid (**L1**) was prepared by condensation of 2‐aminobenzophenone with glyoxylic acid in the presence of ammonium acetate and light irradiation as previously reported.^[41]^ The ligand 4‐phenyl‐*N*‐(prop‐2‐yn‐1‐yl)quinazoline‐2‐carboxamide (**L2**) was synthesized by coupling **L1** as shown in Scheme S1. The complexes [Au(**L2**)(PPh_3_)] (**1**), [Au(**L2**)(TPA)] (**2**) and [Au(**L2**)(Me2‐imy)] (**3**) (Figure 1) were prepared in high yields by reacting the ligand **L2** with [AuCl(PPh_3_)], [AuCl(TPA)] and [AuCl(Me_2_‐imy)] in the presence of diisopropylethylamine (Scheme S2).

The ligand **L2** and complexes **1**–**3** were characterized by multinuclear NMR (^1^H, ^13^C, and ^31^P) and mass spectrometry and their purity was confirmed by elemental analysis (Figures S1–S29 in the Supporting Information). The ^1^H and ^13^C NMR spectra were consistent with the proposed structures of the compounds. Coordination of Au(I) to the alkyne group in **L2** was confirmed by the disappearance of the singlet of the ethynyl hydrogen at 3.13 ppm. The ^31^P NMR spectra of **1** and **2** show a single phosphorus signal at 41.6 and −49.6 ppm, respectively, similarly to other reported gold(I) complexes with alkynyl, PPh_3_ and TPA ligands.[^44^, ^45^] The ν(C≡C) band of **L2** that is observed at 2221 cm^−1^ in the IR spectrum of the free ligand is shifted to lower frequency on coordination of the ethynyl group to Au and appears at 2122 (complex **1**), 2158 (complex **2**), and 2138 cm^−1^ (complex **3**). In the ESI‐MS spectra peaks centered at m/z 288.11 (M+H^+^) (**L2**), 746.16 (M+H^+^) (**1**), 641.15 (M+H^+^) (**2**), and 614.11 (M+Cl^−^) (**3**) were observed with the isotope patterns matching the simulated ones.

### Solution Stability Study

The stabilities of the free ligand **L2** and complexes **1**–**3** were evaluated in PBS (pH 7) containing 1 % DMSO by HPLC. The compounds were found to be stable over 72 h at 37 °C. Moreover, the stabilities of the compounds were monitored in cell culture medium containing 1 % DMSO for 72 h (Figures S30–S33). No changes were observed in the HPLC chromatograms, indicating that the compounds are stable under *in vitro* conditions.

Au(I) has a high affinity for sulfur ligands and while the coordination of Au(I) to S and Se donor atoms in proteins and enzymes plays a role in the mode of action of Au anticancer complexes it can also lead to off‐target binding and deactivation. To model the reaction with biological sulfur ligands the gold complexes were reacted with L‐cysteine on an NMR scale. The ^1^H and ^31^P NMR spectra of the reaction of **1** and **2** with one equivalent L‐cysteine at 37 °C are shown in Figures S34–S37. The ^31^P NMR signals of the coordinated phosphane ligands at 41.6 (complex **1**) and −49.6 ppm (complex **2**) have disappeared after 24 h and the resonances of free PPh_3_ (−5.0 ppm)^[46]^ and free TPA (−96.2 ppm)^[47]^ are visible. The H_α_ and H_β_ protons of L‐cysteine that are observed at 3.92 and 2.94 ppm in the spectrum of the free amino acid, appear at 4.00 and 3.25 ppm which confirms that the phosphane ligands have been replaced by L‐cysteine. The changes in the ^1^H NMR spectrum of **3** (Figure S38) indicate the substitution of the NHC ligand by L‐cysteine. After 24 h a singlet is observed at 8.97 that can be assigned to Me_2_‐imyH^+^ and the H_c_ and H_b_ signals (Figure 1) have shifted from 7.33 and 3.70 ppm to 7.41 and 3.76 ppm, respectively. The H_α_ and H_β_ signals of L‐cysteine at 4.00 and 3.25 ppm confirm the formation of [Au(**L2**)(Cys)]^−^. The signal for the alkyne proton of the free ligand is not observed, when **1**–**3** are reacted with L‐cysteine indicating that **L2** remains bound to Au. The substitution of the NHC ligand of **3** is somewhat surprising, because previous studies of alkynyl NHC Au(I) complexes reported in the literature have shown that thiols displace the more labile alkynyl ligand.[^19^, ^20^] The only exception is (Me)BzImi‐Au(I)(phenylethynyl) that loses the (Me)BzImi ligand ((Me)BzImi=1,3‐dimethylbenzimidazol‐2‐ylidene).^[19]^ Dos Santos et al. studied the ligand exchange reactions of Au(I) NHC complexes with cysteine computationally.^[48]^ They found that the substitution reaction of the 1,3‐Me_2_imy complex had the lowest energy barrier of the 1,3‐R_2_imy series, as the activation energy decreases with decreasing σ‐donor strength and decreasing steric demand of the alkyl substituents.

After longer reaction times (>24 h) insoluble, yellow precipitates formed in all three reactions. This was also the case when an excess of ethanethiol was used as a model for biological sulfur ligands. It was therefore not possible to monitor the potential loss of the alkynyl ligand as the second substitution step.

### XTT Assay

**Figure 2:**
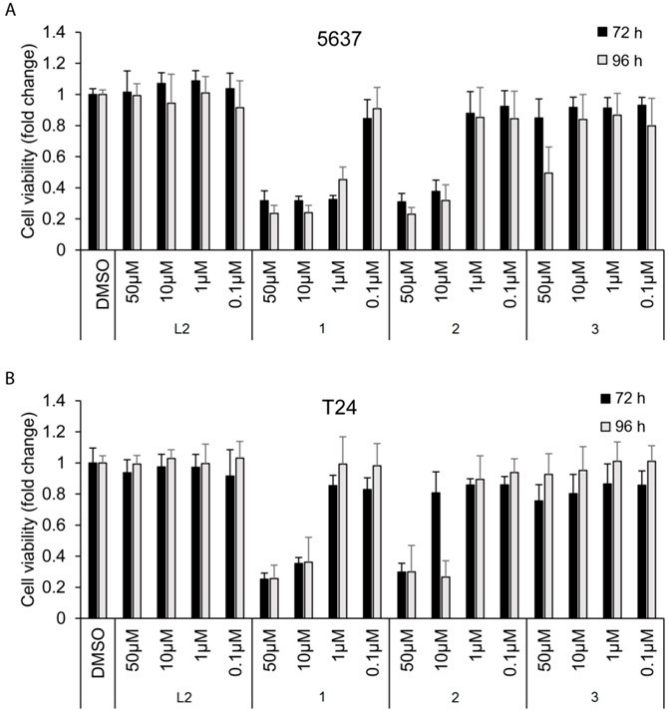
Viable cell number of (A) 5637 and (B) T24 bladder cancer cells following treatment with ligand **L2** and complexes **1**, **2** and **3** or negative control DMSO (carrier). The cell viability was measured post incubation at 72 h and 96 h using the XTT assay. Data are presented as mean±SD (n=3).

The cytotoxicity of the compounds was tested by XTT assay which monitors total viable cell number. The bladder cancer cell lines 5637 and T24 were incubated with each compound at the indicated concentration for 72 and 96 hours (Figure 2). The free ligand had no effect on cell number which was comparable to DMSO control. Complex **1** reduced viable cell numbers for both cell lines by more than 60 % at 10 μM concentration after 72 and 96 hours treatment. Complex **2** also showed similar effects as compound **1** in 5637 cells but showed a later response at 10 μM concentration in T24 cells. This late response is likely due to differences between the two cell lines related to either differences in length of time for the apoptotic process, differences in compound permeability into the cell, or differences in the amount of compound needed to activate apoptosis in the cells or some combination of all these. Complex **3** showed a modest effect on viable cell number only at 50 μM, 96 hour treatment on 5637 cell line but had no effect on T24 cells.

The IC_50_ values of complexes **1** and **2** were calculated from each treatment condition (Table 1). Overall, complexes **1** and **2** had the most significant cytotoxic effects on bladder cancer cell lines.

**Table 1:** IC_50_ (μM) values for complexes **1** and **2** treatment of 5637 and T24 bladder cancer cell lines. All compounds were dissolved in DMSO and added for a 72 h or 96 h incubation period. Data are presented as mean±SE (n=3).

Complex	5637 72 h	96 h	T24 72 h	96 h
**1**	0.17±0.04	0.48±0.06	2.59±0.41	5.29±3.35
**2**	2.56±0.42	2.53±0.55	12.40±11.95	1.71±2.40

### Cellular Uptake

**Figure 3:**
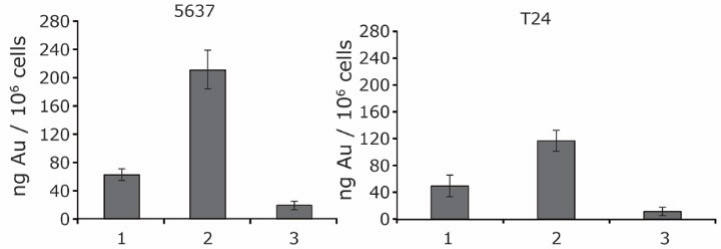
Cellular uptake of Au(I) complexes **1**–**3** in 5637 and T24 cells, incubated for 24 h with the compounds at 5 μM concentration.

In order to evaluate the relationship between cytotoxicity and cellular accumulation, the cellular uptake of the gold complexes into 5637 and T24 cells was quantified using ICP‐MS. Figure 3 shows the intracellular Au levels (as ng of metal per 10^6^ cells) after incubation with 5 μM concentrations of **1**–**3** for 24 h. The most efficient uptake was observed for complex **2** which is in line with the high lipophilicity of the PPh_3_ ligand. The cellular Au levels decreased in the order **2**>**1**>**3**.

### Caspase 3/7 Activity Assay and Western Blotting

**Figure 4:**
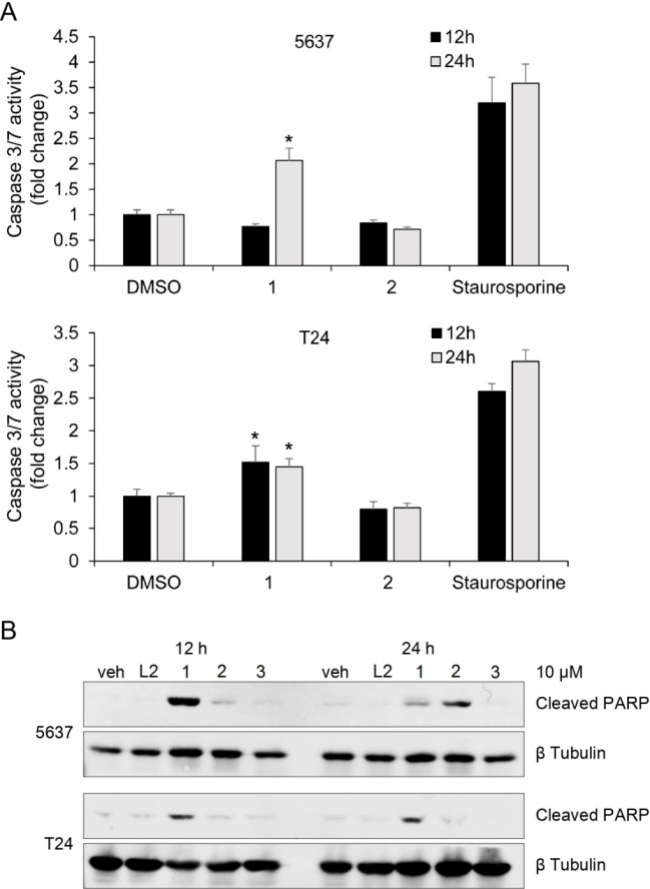
(A) Caspase 3/7 activity of 5637 and T24 bladder cancer cells following treatment with complexes **1**, **2** (10 μM), or negative control DMSO. Caspase 3/7 activity was measured at 12 h and 24 h using the Caspase‐Glo 3/7 assay. Data are presented as mean±SD (n=2). *p<0.05. (B) Increased cleaved PARP in 5637 (top) and T24 (bottom) cell lines in response to complex **1** and **2** (10 μM). Cells were incubated with the indicated compound for 12 h or 24 h followed by cell lysis and Western blot analysis. Blots were probed with β tubulin antibody as a control for protein loading.

Since complexes **1** and **2** showed reduced numbers of viable bladder cancer cells, we determined if these compounds activated apoptotic cell death using a caspase 3/7 activation assay (Figure 4A).

Complex **1** treatment induced activation of caspase 3/7 in both 5637 (>2 fold in 24 h) and T24 (>1.5 in 12 h and >1.4 in 24 h) cell lines. Complex **2** had no effect on caspase 3/7 activity in either cell line at this early time point. Note the full effect on viability was determined at 72 h, but to have enough cells to do the caspase3/7 assay we opted for an earlier timepoint before the cells had died. Thus, the difference between compound **1** and **2** in the caspase assay is most likely due to differences in access and inhibition time of the compounds rather than a different mechanism with compound **2** less effective in this assay at these early timepoints. A second marker of caspase3‐mediated apoptosis, cleaved PARP protein level, was also measured in both 5637 and T24 cell lines following incubation at the indicated time points (Figure 4B). Complex **1** showed increased cleaved PARP within 12 hours while complex **2** showed increased cleaved PARP in 5637 cells only after 24 hours, indicating both compounds activate caspase activity although with different timing.

### Thioredoxin Reductase Assay

**Figure 5:**
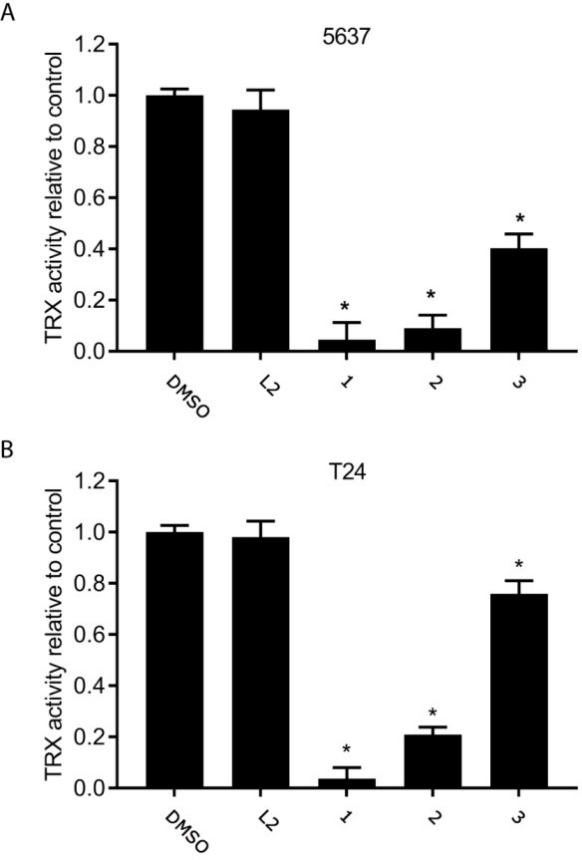
TrxR activity in (A) 5637 and (B) T24 bladder cancer cells after treatment with **1**–**3** and **L2** at 5 μM concentration for 12 h. The values indicate the fold change of TrxR activity relative to DMSO treated cells. Analysis was done on 30 μg cell lysate protein. Data are presented as mean±SD (n=3). *p<0.05.

Intracellular anti‐oxidant states are often dysregulated in chemo‐resistant cancers. Overexpression of thioredoxin reductase (TrxR), an enzyme that controls the intracellular redox state, is a key element for the survival of drug‐resistant cancer cells.^[49]^ Gold complexes are known inhibitors of TrxR. The inhibition of TrxR can lead to a decrease in mitochondrial thiols and to an increase in reactive oxygen species (ROS) which in turn can affect the mitochondrial membrane permeability and trigger apoptosis.^[50]^ Therefore, we measured the activity of TrxR in 5637 and T24 cell lines following incubation with each of the three Au(I) complexes and the free ligand **L2** (Figure 5).

After 12 hours of incubation at 5 μM, 5637 and T24 TrxR activity is significantly decreased by complex **1** (>90 %) and **2** (>80 %). Although complex **3** has minor effects on cytotoxicity, it also showed a significant decrease in TrxR activity in 5637 cells (60 %) and modest decrease in T24 cells (25 %). This indicates that the Au complexes are active in modulating ROS balance, leading to an ROS imbalance and resulting in apoptosis.

### TSPO Interaction – in Silico Molecular Docking

Complexes **1**–**3** were designed to contain an alkynyl‐functionalized derivative of a TSPO binder. The published structure‐affinity relationships of 4‐phenylquinazoline carboxamides suggest that the bulky Au(phosphane) entities would hinder the interaction of **1** and **2** with TSPO.^[41]^ However, if ligand substitution occurs inside the cell, the free ligand may bind to TSPO. The TrxR inhibitory activity of Au(I) complexes is attributed to the binding of Au(I) to the C‐terminal redox active Cys‐Sec center following ligand exchange. Meyer et al. studied the reaction of alkynyl phosphane Au(I) complexes with a selenocysteine‐containing model tetrapeptide by mass spectrometry and observed peaks corresponding to the coordination of a “naked” Au atom as well as peaks corresponding to the coordination of Au(alkynyl) and Au(phosphane) entities to the peptide indicating that the reaction with TrxR can lead to the cleavage of the Au alkynyl bond.^[21]^

**Figure 6:**
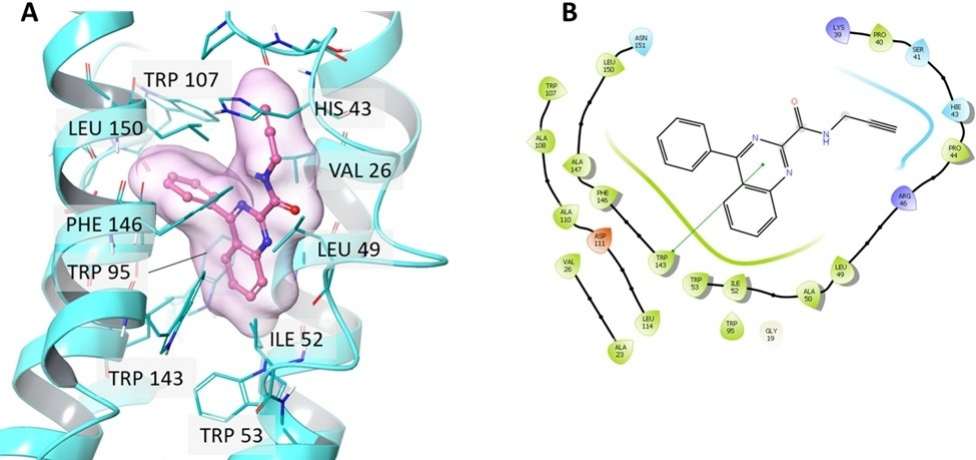
Molecular docking of **L2** to TSPO. A. Predicted docking pose of **L2** in the TSPO binding site (PDB 2MGY). The protein is rendered as ribbons with transmembrane helices shown in cyan. The residues in the binding pocket are shown as sticks (cyan) and the ligand as a ball and stick representation (purple). B. 2D ligand interactions of **L2** created using the Schrödinger Maestro program. The π‐stacking interaction with Trp 143 is shown as a green line.

Molecular docking showed that if **L2** is released on the formation of a coordinative bond between Au(I) and the active site of TrxR, the free ligand fits tightly into the binding site of TSPO. The free ligand **L2** had a Glide docking score of −8.89 kcal/mol (Figure 6). Moreover, the docked structure showed a high resemblance to the native PK11197 binding mode (PDB 2MGY).^[50]^ The orientation of the 4‐phenylquinazoline ring structure of **L2** in the binding site is similar to that of the 3‐phenylisoquinoline ring of the established TSPO binder PK11197 (Figure 6). The core structure of **L2** forms a π‐stacking interaction with Trp 143 and favorable contacts with Ala 23, Val 26, Ile 52, Trp 53, Trp 95, Trp 107, Leu 114 and Leu 150 (Figure 6B). Furthermore, the carboxamide group of **L2** is engaged in additional hydrophobic interactions with Pro 40, Phe 146, and Leu 150. Collectively, the docking investigation showed that compound **L2** has a strong predicted binding affinity to the TSPO binding site. The binding of **L2** to TSPO is achieved predominantly via hydrophobic contacts with the interaction profile largely similar to that of the known PK11195 compound.^[51]^

## Conclusion

The alkynyl phosphane Au(I) complexes **1** and **2** and the alkynyl NHC Au(I) complex **3** inhibit cellular TrxR activity. **1** and **2** are also potent cytotoxins while in the case of **3** the TrxR inhibition does not translate into cytotoxic activity. The cytotoxicity is due to the presence of Au(I), as the free quinazoline carboxamide is inactive. **1** and **2** appear to reduce cell viability by inducing ROS and caspase‐dependent apoptotic cell death. Thus, **1** and **2** add to the small number of cytotoxic Au(I) alkynyl phosphane complexes.

## Experimental Section

**Materials and Instrumentation**: All chemicals were obtained from commercial sources unless stated otherwise. [Au(PPh_3_)Cl],^[52]^ [Au(TPA)Cl] (TPA=1,3,5‐triaza‐7‐phosphaadamantane),^[53]^ [AuCl(Me_2_‐imy)] (Me_2_‐imy=1,3‐dimethylimidazol‐2‐ylidene)^[54]^ were synthesized as previously reported. 4‐Phenylquinazoline‐2‐carboxylic acid (**L1**) was prepared according to the literature procedure with slight modifications (Supporting Information).^[41]^ ^1^H, ^13^C and ^31^P NMR spectra were recorded on a Varian 500 AR spectrometer using a 500 MHz Smart Probe. The data were processed using MestreNova with a 300 Hz line broadening and backward linear prediction. Mass spectra were measured using a Waters LCT Premiere XE with electron spray ionisation and time of flight mass analyzer. Elemental analysis (C, H and N) were performed by an Exeter Analytical CE‐440. FT‐IR spectra were recorded on a PerkinElmer FT‐IR spectrometer fitted with an ATR accessory. The HPLC studies were carried out using an Agilent 1200 series DAD analytical HPLC instrument. Inductively coupled plasma mass spectrometry (ICP‐MS) measurements for Au were performed on a PerkinElmer ELAN DRCe, ICP‐MS (Waltham, USA) equipped with a flow injection autosampler (FIAS 93 plus) in standard mode in a class 1000 (ISO class 6) cleanroom. Instrumental operating conditions were the following: ICP RF Power 1250 W: plasma gas flow 15 L min^−1^, auxiliary gas flow 1 L min^−1^, nebulizer gas flow 1.04 L min^−1^, observed isotope ^197^Au. Calibration standard solutions were prepared from a single element standard (Inorganic Ventures, 1000 μg mL^−1^) prepared in Milli‐Q™ water (18.3 mΩ) (Millipore, Bedford, USA) with 1 % HNO_3_ (ROMIL‐SpA™, USA). Indium (^115^In) was used as internal standard to account for instrumental drift and matrix effects, and method blanks, duplicate samples and aqua checks were incorporated in the analysis. The calibration curve was obtained using known concentrations of Au standard solutions (0–50 ppb).

**Synthesis of 4‐phenyl‐*N*‐(prop‐2‐yn‐1‐yl)quinazoline‐2‐carboxamide, L2**: To **L1** (252 mg, 1 mmol) in dry dichloromethane (10 mL) was added oxalyl chloride (175 μL, 2 mmol) and DMF (3 drops). The solution was stirred at room temperature for 100 min and evaporated in a rotary evaporator. The residue was dissolved in dry THF (10 mL) and propargylamine (80 μL, 1.2 mmol) and triethylamine (700 μL, 5 mmol) in dry THF (10 mL) were added at 0 °C. After 15 min., the resulting solution was stirred at room temperature for 6 h and evaporated using a rotary evaporator. The residue was dissolved in ethyl acetate (30 mL), washed with brine (2×10 mL), and dried over MgSO_4_ and finally concentrated by rotary evaporation. Silica gel column chromatography was used to purify the crude compound using ethyl acetate: petroleum ether (3: 7) as the mobile phase. A pale yellow solid was obtained (275 mg, 96 %). Anal. Calc. (%) for C_18_H_13_N_3_O (287.10): C, 75.25; H, 4.56; N, 14.63. Found (%): C, 75.20; H, 4.55; N, 14.59. ESI‐MS: 288.1178 [M+H]^+^. ^1^H NMR (DMSO‐*d*_6_): δ 9.37 (t, 1H, H−NH, *J*=5.0 Hz), 8.09–8.20 (m, 3H, H−Ar), 7.78–7.85 (m, 3H, H−Ar), 7.62–7.63 (m, 3H, H−Ar), 4.14 (dd, 2H, C*H*_2_, *J*=5.0 Hz), 3.13 (s, 1H, C≡C*H*). ^13^C NMR (DMSO‐*d6*): δ 168.7 (C=O), 163.2 (Ar), 154.0 (Ar), 150.8 (Ar), 136.6 (Ar), 135.3 (Ar), 130.6 (Ar), 130.0 (Ar), 129.5 (Ar), 129.0 (Ar), 127.3 (Ar), 122.6 (Ar), 81.5 (CCH), 73.2 (CCH), 29.1 (CH_2_). IR (cm^−1^): 3325 m, 3285 m, 2920 w, 2221 (C≡C) w, 1672 s (C=O), 1611 w, 1561 m, 1556 s, 1448 m, 1391 m, 1337 m, 1262 w, 1201 w, 1167 w, 1131 w, 1072 w, 1024 w, 981 w, 927 w, 794 m, 768 s, 719 w, 671 s.

**Synthesis of [Au(L2)(PPh_3_)] (1)**: *N,N*‐diisopropylethylamine (DIPEA) (52.3 μL, 0.3 mmol) and ligand **L2** (28.7 mg, 0.1 mmol) were added to [AuCl(PPh_3_)] (49.5 mg, 0.1 mmol) in dichloromethane (10 mL). After stirring at room temperature for 72 h the reaction mixture was evaporated to dryness. The residue was subjected to flash chromatography on SiO_2_ (eluent: CHCl_3_/MeOH/Et_3_N, 60 : 3 : 0.5). The analytically pure product **1** was obtained as a yellow solid after recrystallization from a dichloromethane/n‐hexane (10 : 1) mixture (69.3 mg, 93 % yield). Anal. Calc. (%) for C_36_H_27_AuN_3_OP (745.15): C, 57.99; H, 3.65; N, 5.64. Found (%): C, 57.95; H, 3.64; N, 5.59. ESI‐MS: 746.1627 [M+H]^+^. ^1^H NMR (DMSO‐*d*_6_): δ 9.10 (t, 1H, N*H*, *J*=5.0 Hz), 8.09–8.22 (m, 3H, H−Ar), 7.80–7.87 (m, 3H, H−Ar), 7.46–7.65 (m, 18H, H−Ar), 4.16 (m, 2H, C*H*_2_). ^13^C NMR (DMSO‐*d6*): δ 168.7 (C=O), 162.7 (Ar), 154.3 (Ar), 150.8 (Ar), 136.7 (Ar), 135.3 (Ar), 134.2 (Ar), 132.3 (Ar), 130.6 (Ar), 129.9 (Ar), 129.4 (Ar), 129.0 (Ar), 127.3 (Ar), 122.6 (Ar), 66.3 (CCH), 30.3 (CH_2_). ^31^P{^1^H}−NMR (243 MHz, DMSO): δ 41.60. IR (cm^−1^): 3142 w, 3071 w, 2122 (C≡C) w, 1682 m (C=O), 1613 w, 1563 m, 1538 w, 1506 m, 1486 m, 1438 m, 1389 m, 1351 m, 1312 w, 1232 w, 1205 w, 1105 w, 1074 w, 988 w, 780 m, 751 m, 662 m, 691 s.

**Synthesis of [Au(L2)(TPA)] (2)**: The synthesis was carried out as described for **1**, with [AuCl(TPA)] (40 mg, 0.1 mmol) instead of [Au(PPh_3_)Cl]. The residue was subjected to flash chromatography on SiO_2_ (eluent: CHCl_3_/MeOH/Et_3_N, 50 : 2 : 0.5). The analytically pure product **2** was obtained after recrystallization from a dichloromethane/n‐hexane (9 : 1) mixture (62 mg, 96 %). Anal. Calc. (%) for C_24_H_24_AuN_6_OP (640.14): C, 45.01; H, 3.78; N, 13.12. Found (%): C, 44.99; H, 3.79; N, 13.08. ESI‐MS: 641.1539 [M+H]^+^. ^1^H NMR (DMSO‐*d*_6_): δ 9.02 (t, 1H, N*H*, *J*=5.0 Hz), 8.10–8.28 (m, 3H, H−Ar), 7.80–7.87 (m, 4H, H−Ar), 7.64 (m, 2H, H−Ar), 4.21–4.44 (m, 12H, C*H*_2_ (TPA)), 4.08 (m, 2H, C*H*_2_ (**L2**)). ^13^C NMR (DMSO‐*d6*): δ 168.6 (C=O), 162.6 (Ar), 154.2 (Ar), 150.8 (Ar), 136.7 (Ar), 135.3 (Ar), 130.7 (Ar), 130.0 (Ar), 129.5 (Ar), 129.0 (Ar), 127.3 (Ar), 122.6 (Ar), 99.7 (CCH), 72.2 (CH_2_ (PTA)), 51.3 (CH_2_ (PTA)), 30.4 (CH_2_ (**L2**)). ^31^P{^1^H}−NMR (243 MHz, DMSO): δ −49.60. IR (cm^−1^): 3142 w, 2932 w, 2158 (C≡C) w, 1682 s (C=O), 1613 w, 1563 m, 1506 s, 1488 s, 1438 w, 1389 m, 1353 m, 1284 w, 1240 w, 1203 w, 1103 w, 1012 w, 969 w, 852 w, 780 w, 741 w, 675 m, 697 s.

**Synthesis [Au(L2)(Me_2_‐imy)] (3)**: The synthesis was carried out as described for **1**, with [AuCl(Me_2_‐imy)] (33 mg, 0.1 mmol) instead of [Au(PPh_3_)Cl]. The residue was subjected to flash chromatography on SiO_2_ (eluent: CHCl_3_/MeOH/Et_3_N, 65 : 2.5 : 0.5). The analytically pure product **3** was obtained after recrystallization from a dichloromethane/n‐hexane (8 : 2) mixture (47 mg, 81 %). Anal. Calc. (%) for C_23_H_20_AuN_5_O (579.13): C, 47.68; H, 3.48; N, 12.09. Found (%): C, 47.62; H, 3.45; N, 12.07. ESI‐MS: 614.1190 [M+Cl]^−^. ^1^H NMR (DMSO‐*d*_6_): δ 8.98 (t, 1H, N*H*, *J*=5.0 Hz), 8.11–8.23 (m, 3H, H−Ar), 7.81–7.88 (m, 3H, H−Ar), 7.63–7.65 (m, 3H, H−Ar), 7.33 (s, 2H, H‐c), 4.13 (s, 2H, C*H*_2_ (**L2**)), 3.70 (s, 6H, H‐b). ^13^C NMR (DMSO‐*d6*): δ 186.1 (C‐c), 168.7 (C=O), 162.6 (Ar), 154.2 (Ar), 150.8 (Ar), 136.7 (Ar), 135.3 (Ar), 130.6 (Ar), 130.0 (Ar), 129.5 (Ar), 129.0 (Ar), 127.3 (Ar), 123.0 (Ar), 122.6 (Ar), 99.6 (CCH), 37.9 (C‐b), 30.74 (CH_2_ (**L2**)). IR (cm^−1^): 3142 w, 3107 w, 2928 w, 2849 w, 2138 w (C≡C), 1690 s (C=O), 1611 w, 1559 m, 1532 m, 1498 s, 1484 s, 1438 w, 1385 m, 1349 s, 1224 w, 1169 w, 1137 w, 1074 w, 1012 w, 998 w, 824 m, 778 s, 743 w, 697 s.

**Reaction of Complexes 1—3 with L‐cysteine**: Complex **1** (7.45 mg, 0.01 mmol), **2** (6.4 mg, 0.01 mmol) or **3** (5.79 mg, 0.01 mmol) and L‐cysteine (12.2 mg, 0.1 mmol) were dissolved in DMSO‐*d*_6_/D_2_O (1 : 1) and kept at 37 °C for 24 h. The reaction was monitored by ^1^H and ^31^P NMR spectroscopy.

**Stability Study**: The stabilities of **L2** and complexes **1**–**3** were tested by dissolving the compounds in PBS/1 % DMSO or DMEM (Dulbecco's Modified Eagle's Medium – high glucose)/1 % DMSO and keeping them for 3 d at 37 °C. The solutions were analyzed by HPLC using a Phenomenex Luna C18 (5 μm, 100 Å, 250 mm×4.60 mm i.d.) column at a flow rate of 0.5 mL/min with 230 nm and 280 nm UV detection at room temperature. The mobile phase was 70 : 30 acetonitrile (0.1 % trifluoroacetic acid) : water (0.1 % trifluoroacetic acid).

**Computational Modeling Methods**: In silico molecular docking was performed using the Schrödinger software suite implemented using the Maestro graphical user interface.^[55]^ The NMR structure of TSPO in complex with the small molecule ligand PK11195 (PDB 2MGY) was retrieved from the protein data bank.^[56]^ Schrödinger's ‘protein preparation wizard’ workflow was used to prepare the PDB structure for docking.^[57]^ Briefly, hydrogen atoms were added, missing side chains and loops were filled using the Prime module of Schrödinger, hydrogen bonds were optimized and energy minimization was performed with OPLS3 force field as a final step.^[58]^ The prepared protein structure was used as an input for the receptor grid generation tool in Maestro. The coordinates of the PK11195 were used as a reference position for receptor grid generation. The maximum length of ligands to be docked was set to 20 Å. The small molecule compound **L2** structure was initially drawn using the ChemDraw (PerkinElmer Informatics) application and later prepared for molecular docking using the LigPrep module in the Schrödinger suite.^[56]^ All possible protonation and ionization states were generated at pH 7.0 and resulting low energy 3D structures were saved as ‘Maestro’ format for docking.

Glide (Grid‐based Ligand Docking with Energetics) program in Schrödinger was used for the docking studies.^[57]^ The ‘extra precision’ (XP) setting of Glide was chosen and ligand sampling was enabled for docking procedure. Other settings of Glide were set to defaults. Finally, the top 10 poses based on the highest docking score were generated.^[59]^ The ligand poses were individually visualized and rendered using the Maestro program.

**Cell Culture**: Human bladder cancer cell lines 5637 and T24 were a gift from Dr. Rosser's lab (University of Hawaii Cancer Center). Cells were cultured in RPMI‐1640 (Corning) supplemented with 10 % fetal bovine serum (Seradigm) and 1 % Antibiotic‐Antimycotic (ThermoFisher Scientific). Cells were incubated in humidified atmosphere containing 5 % CO_2_ at 37 °C.

**XTT Assay**: Cells were seeded in 96‐well culture plates at a concentration of 5×10^3^ cells/well. The next day, compounds **1**, **2**, **3** and **L2** were each tested independently at 100 nM, 1 μM, 10 μM and 50 μM concentration for 72 hours. Cell viability was then assessed by using XTT kit (Biotium) according to the manufacturer's protocol and incubated at 37 °C for 3 h. The absorbance was measured by ELISA plate reader (Perkin Elmer Envision) at 500 nm with a reference wavelength at 650 nm. The IC_50_ value of each compound was calculated as the concentration reducing the proliferation of the cells by 50 % and is presented as mean (±S.E) of three independent experiments.

**Cellular Uptake**: 5×10^3^ cells/mL 5637 and T24 cells were seeded in T25 cm^2^ flasks. After overnight incubation, the medium was replaced, and the cells were treated with the respective Au complex (5 μM) for 24 h. The cells were washed twice with cold PBS, harvested by trypsinization and counted with a hemocytometer. The samples were digested in a closed vessel microwave digestion system (AntonPaar Multiwave 3000, Graz, Austria) as described in reference [60] using trace metal grade oxidizing agents HNO_3_ (ROMIL‐SpA™, USA) and H_2_O_2_ (TraceSELECT® Ultra SIGMA‐ALDRICH, USA). After cooling, the samples were diluted with Milli‐Q water (18.3 mΩ Milli‐Q Element system™, Merck Millipore, Carrigtwohill, Co. Cork, Ireland) so that the final concentration of HNO_3_ was 1 %. Each mineralized sample was filtered, and the Au content was determined by ICP‐MS as described above.

**Caspase 3/7 Activity Assay**: Cells were seeded in a 96 well plate and incubated with each compound for 12 and 24 h at 10 μM concentration. For detection of apoptosis, 100 μL of Caspase‐Glo 3/7 DEVD‐aminoluciferin substrate solution (Promega) was added to each well and the plates were incubated for 30 min. at room temperature. Caspase 3/7‐activity was detected by measuring the luminescence signal using an ELISA plate reader (Perkin Elmer Envision).

**Western Blotting**: Cells were seeded in 6 well plates and treated with 10 μM of each compound independently for 12 and 24 h. Cells were then washed once with PBS and lysed with a RIPA lysis buffer containing protease inhibitor cocktail (Roche). All protein samples were resolved by 10 % SDS‐PAGE after boiling for 5 min in SDS sample buffer. The resolved proteins were transferred to a nitrocellulose membrane and subsequently analyzed by immunoblot using antibodies specific for cleaved PARP (Cell Signaling Technology) and β tubulin (Proteintech).

**Thioredoxin Reductase Assay**: Cells were seeded on a 100 mm culture dish overnight prior to 12 h treatment with **1**, **2**, **3** or **L2** at 5 μM concentration. Cells were washed 3 times with cold PBS and lysed with RIPA lysis buffer containing protease inhibitor cocktail (Roche). The cleared protein lysate concentration was measured using a Pierce BCA Protein Assay Kit (ThermoFisher Scientific). Equal amounts of protein lysates were used to detect thioredoxin reductase activity according to the manufacturer's protocol (Sigma). A thioredoxin reductase specific inhibitor was used to inhibit thioredoxin reductase to detect isolate and measure only changes in thioredoxin reductase activity. Lysates were tested in a 96 well plate as duplicates. The yellow color formation in each sample was measured at an absorption of 412 nm using an ELISA plate reader (Perkin Elmer Envision). The activity was calculated based on the amount of TNB (5‐thio‐2‐nitrobenzoic acid) produced per minute per mg of total protein corrected for the difference between the presence of the TrxR inhibitor and total activity.

## Conflict of interest

The authors declare no conflict of interest.

## Supporting information

As a service to our authors and readers, this journal provides supporting information supplied by the authors. Such materials are peer reviewed and may be re‐organized for online delivery, but are not copy‐edited or typeset. Technical support issues arising from supporting information (other than missing files) should be addressed to the authors.

Supplementary
