# Supplementary material for: Gold(I) Complexes with a Quinazoline Carboxamide Alkynyl Ligand: Synthesis, Cytotoxicity, and Mechanistic Studies
Source: Eur J Inorg Chem. 2021 May 11;2021(20):1921–8. doi: 10.1002/ejic.202100120 (PMC8252463; doi:10.1002/ejic.202100120)
Supplement: Supplementary file 1 — Supplementary [file EJIC-2021-1921-s001.pdf]

European Journal of Inorganic Chemistry

Supporting Information

Gold(I) Complexes with a Quinazoline Carboxamide Alkynyl Ligand: Synthesis, Cytotoxicity, and Mechanistic Studies

Leila Tabrizi[+], Won Seok Yang[+], Chetan Chintha, Liam Morrison, Afshin Samali, Joe W. Ramos,* and Andrea Erxleben*

Synthesis of 4-phenylquinazoline-2-carboxylic acid, L1

A solution of glyoxylic acid (10.0 mmol, 0.741 g) in water (10 mL) was added dropwise to a solution of 2-aminobenzophenone (10.0 mmol, 1.972 g) and ammonium acetate (30.0 mmol, 2.312 g) in absolute ethanol (30 mL). The mixture was stirred at room temperature for 10 min. After addition of 20 mL of water, the orange solid was collected by filtration and washed with absolute ethanol and diethyl ether to give 1,2-dihydro-4-phenylquinazoline-2-carboxylic acid which was dissolved in DMF (15 mL) and allowed to stand for 18 h under external light irradiation with a 20 W halogen tungsten lamp. After addition of 15 mL of water, the mixture was cooled in an ice bath for 24 h and the yellow precipitate formed was collected by filtration and washed with water (3 mL) and diethyl ether (5 mL) to give 4-phenylquinazoline-2-carboxylic acid which was recrystallized from acetonitrile/H_2O (8:3) (yield: 1.825 g, 73%). ^{1}H NMR ($CDCl_3$): δ 8.33 (t, 1H, H-Ar, ^3J = 10 Hz), 8.25 (t, 1H, H-Ar, ^3J = 10 Hz), 8.03-8.08 (m, 1H, H-Ar), 7.75-7.82 (m, 3H, H-Ar), 7.58-7.63 (m, 3H, H-Ar). ^{13}C NMR ($CDCl_3$): δ 169.7, 162.8, 151.1, 150.2, 135.2, 130.3, 130.2, 129.8, 128.8, 127.5, 123.3. ESI-MS: 251.0821 $[M + H]^+$.

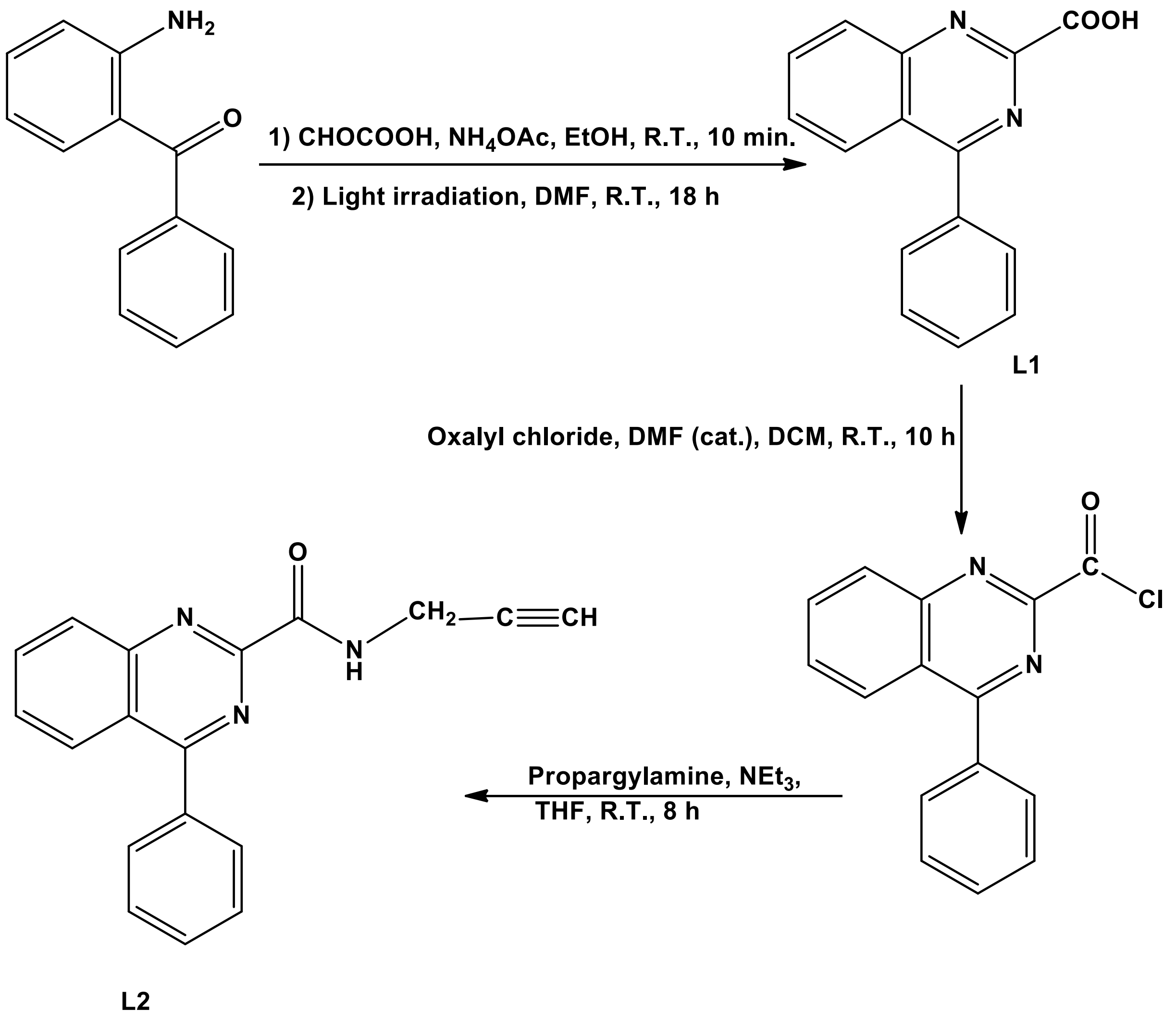

Scheme S1. Synthetic pathway of **L1** and **L2**.

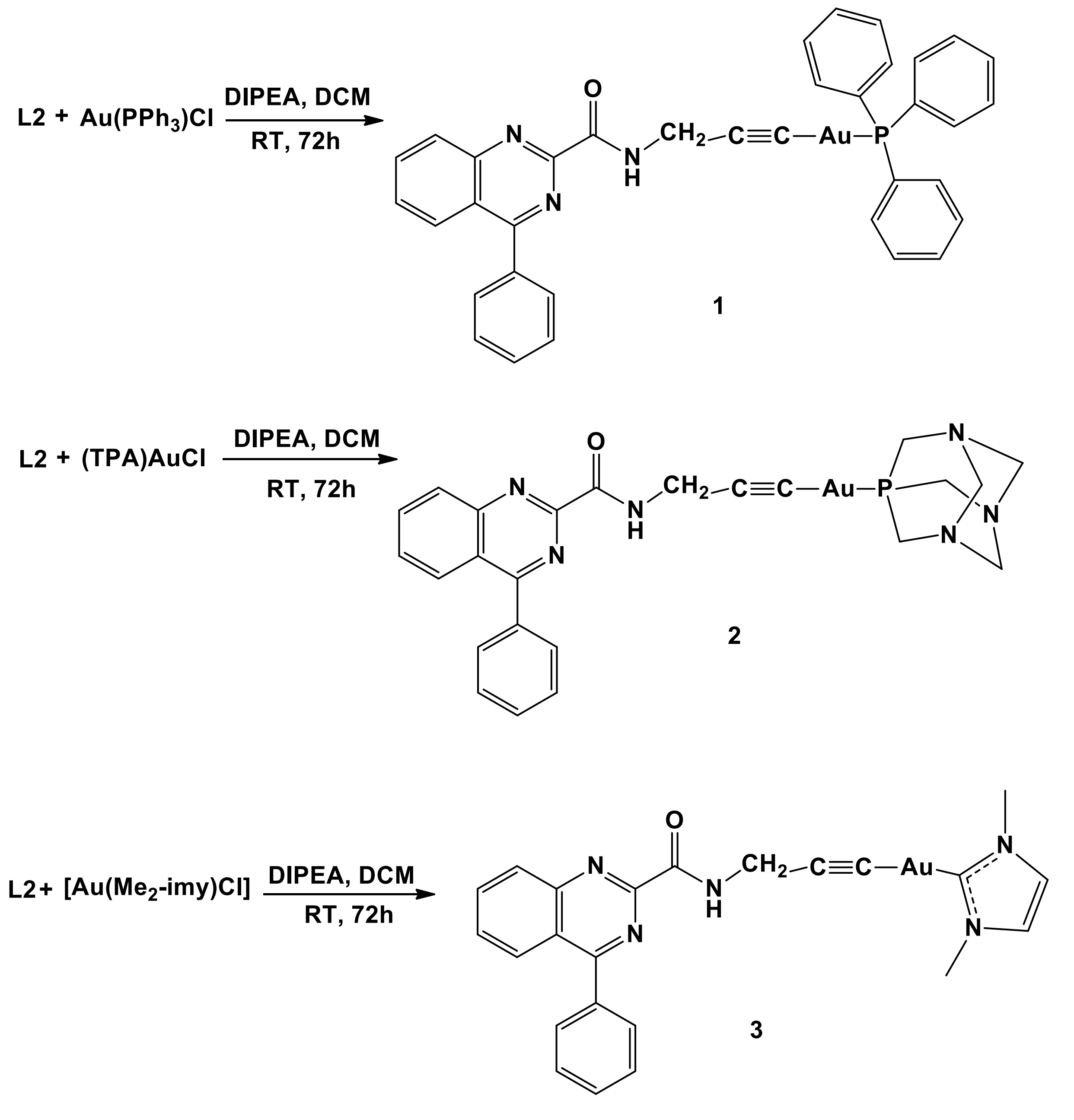

Scheme S2. Synthetic pathway of complexes **1**-**3**.

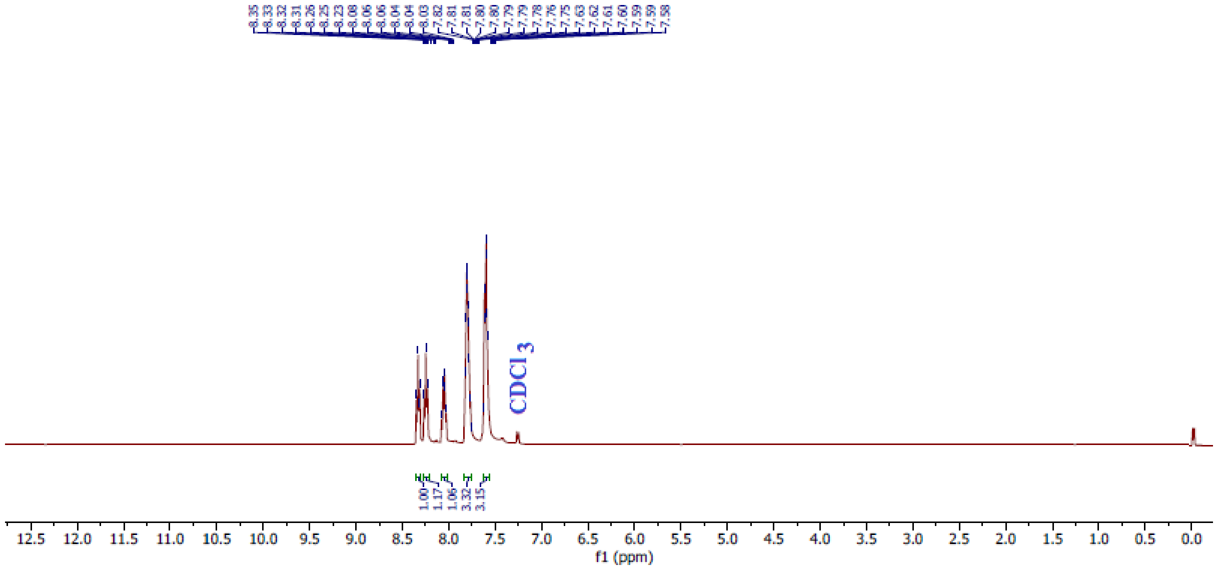

Figure S1. ^{1}H NMR spectrum of **L1** ($CDCl_3$).

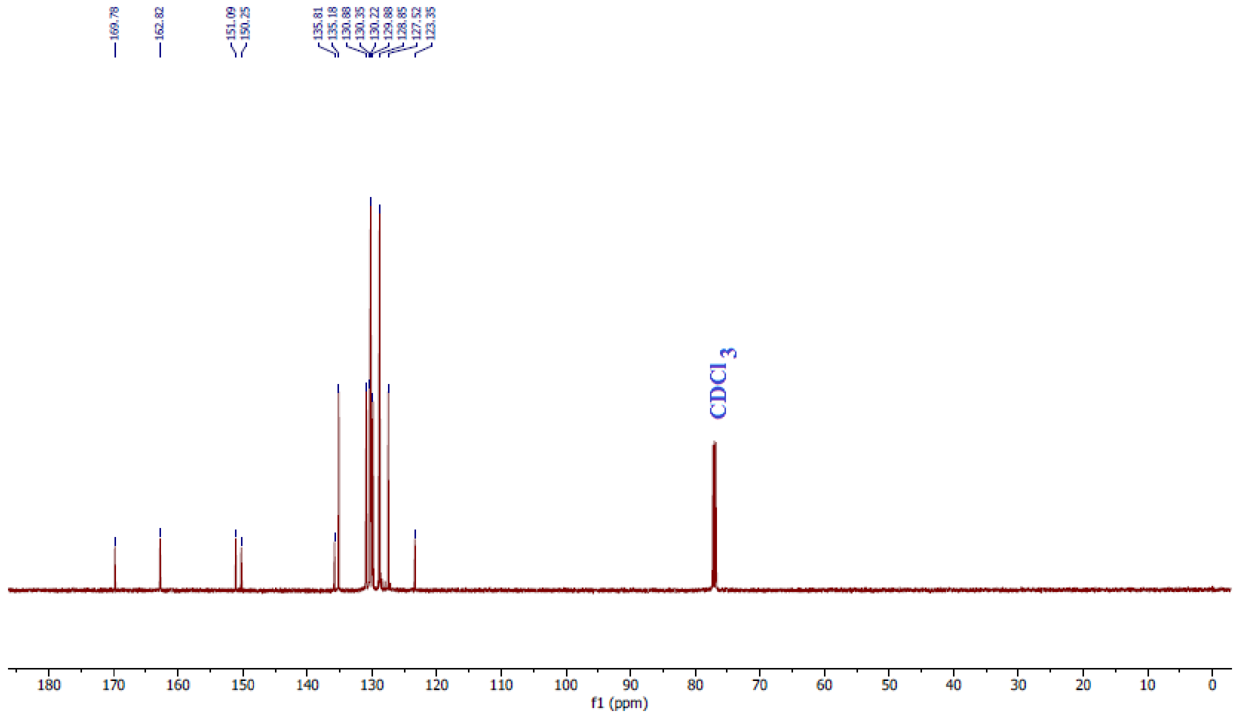

Figure S2. ^{13}C NMR spectrum of ligand **L1** ($CDCl_3$).

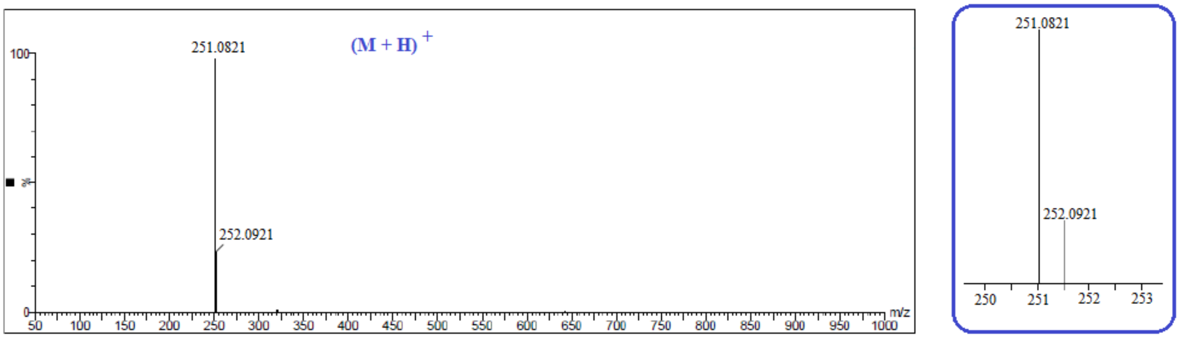

Figure S3. ESI-MS spectrum of **L1**.

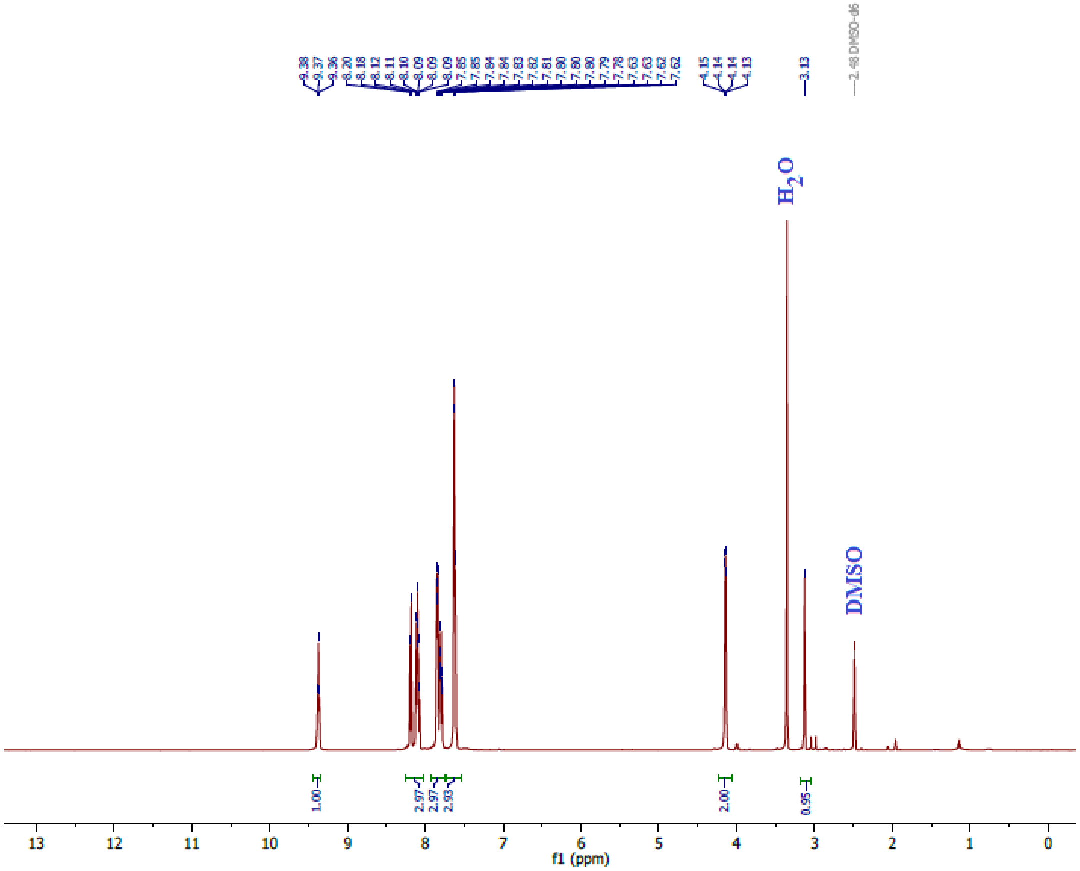

Figure S4. ^{1}H NMR spectrum of **L2** (DMSO-d_6).

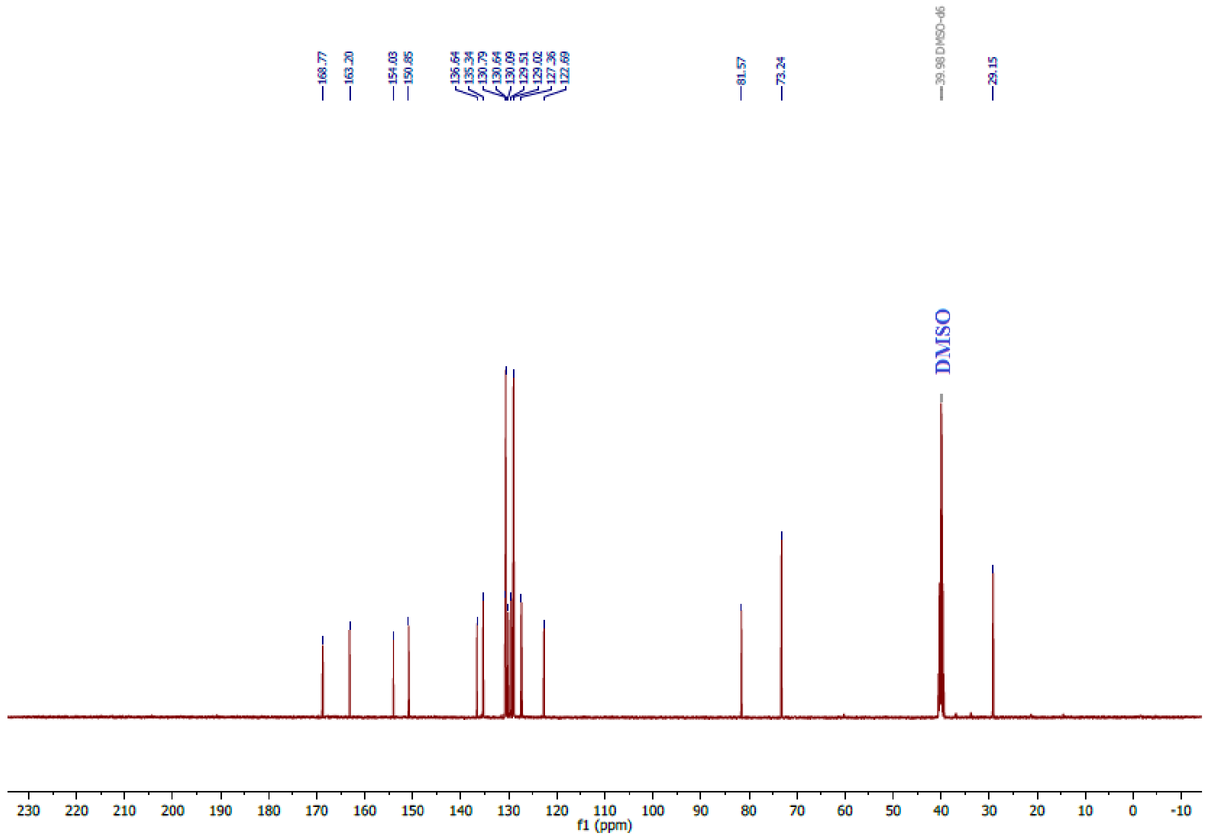

Figure S5. ^{13}C NMR spectrum of **L2** (DMSO-d_6).

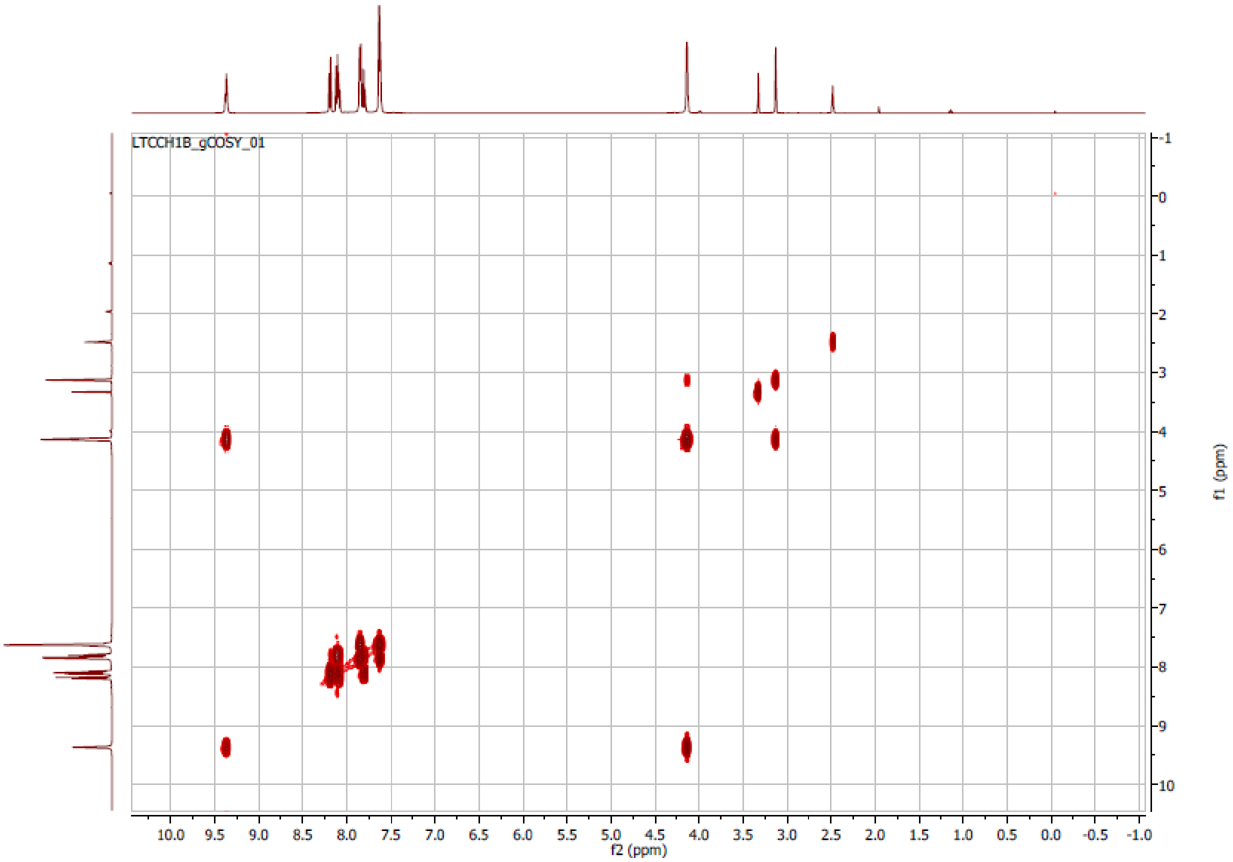

Figure S6. COSY NMR spectrum of **L2** (DMSO-d_6).

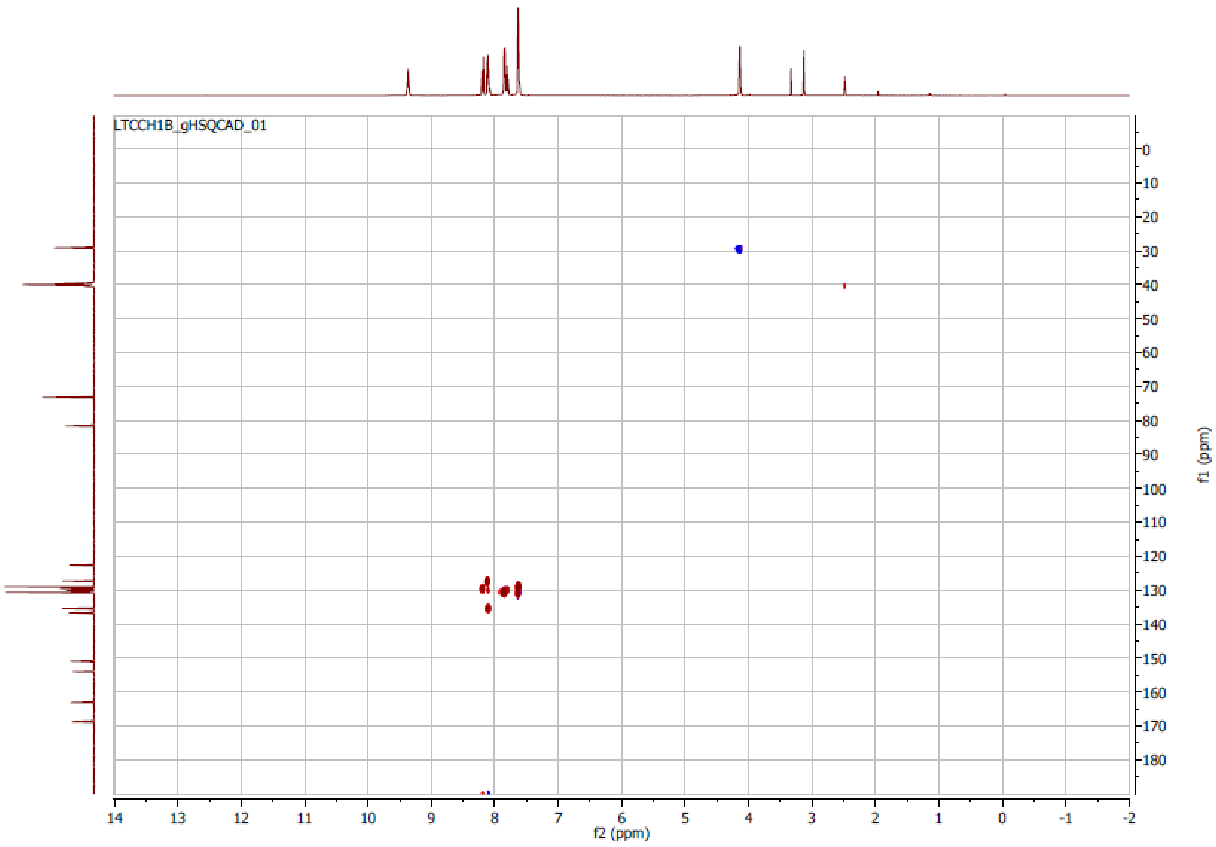

Figure S7. HSQCAD NMR spectrum of **L2** (DMSO-d_6).

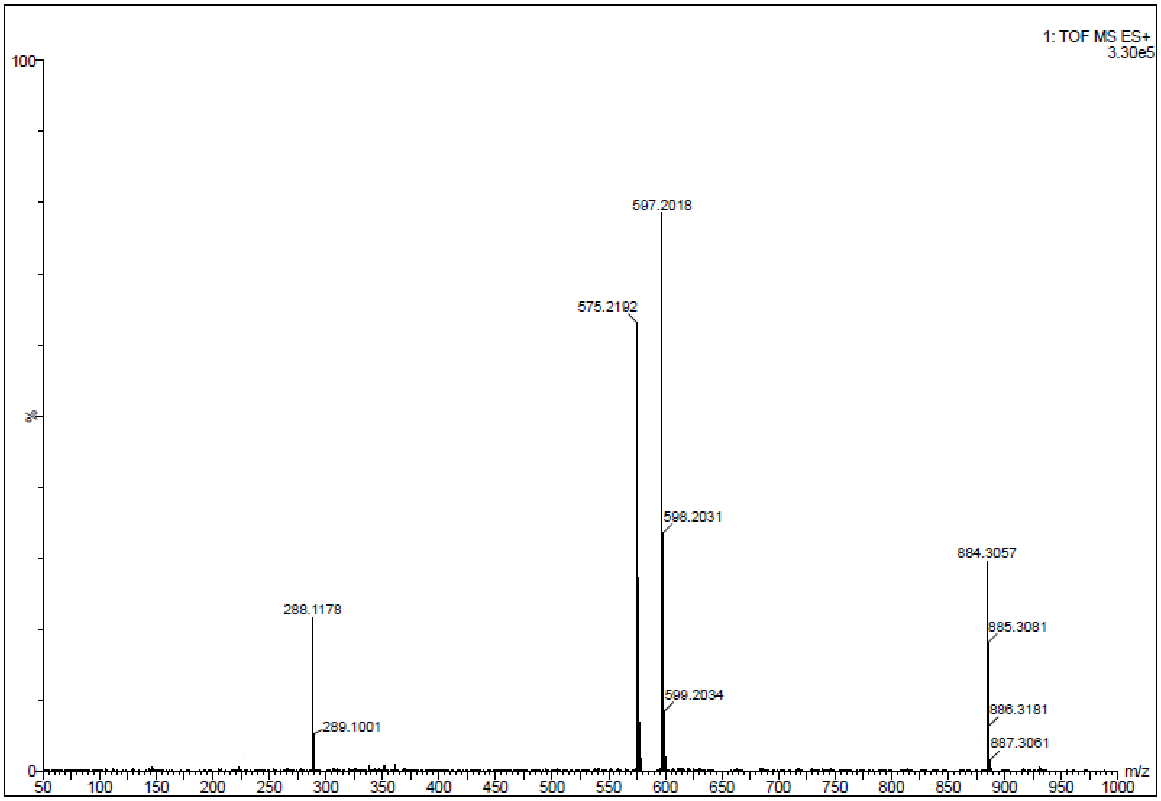

Figure S8. ESI-MS spectrum of **L2** (288 ($M+H^{+}$), 575 ($2M+H^{+}$), 597 ($2M+Na^{+}$), 884 ($3M+Na^{+}$)).

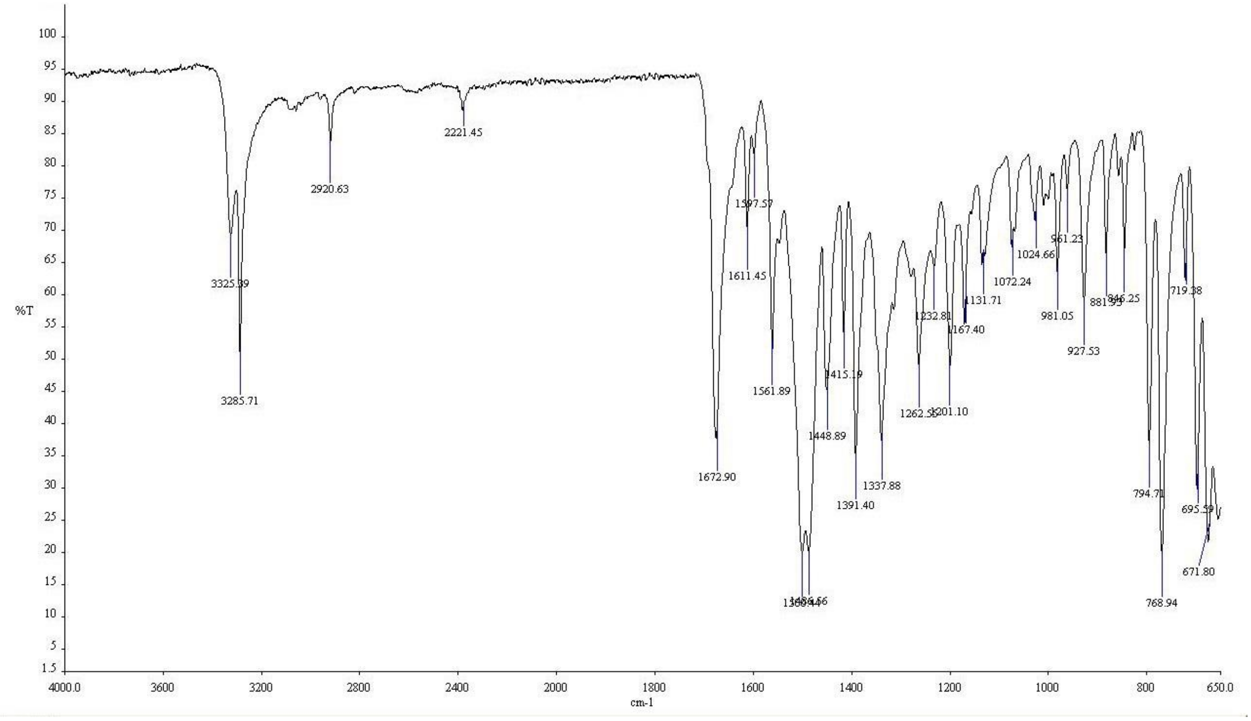

Figure S9. IR spectrum of **L2**.

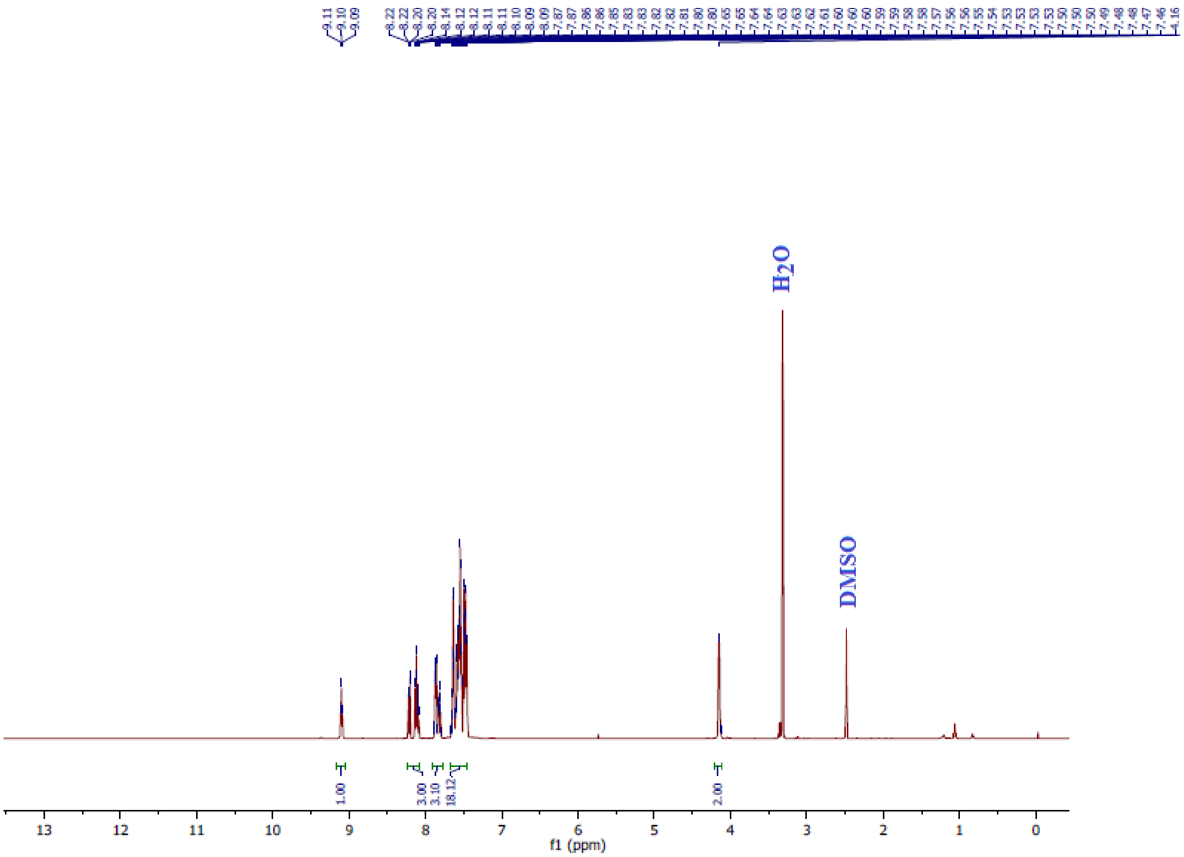

Figure S10. ^{1}H NMR spectrum of complex **1** (DMSO-d_6).

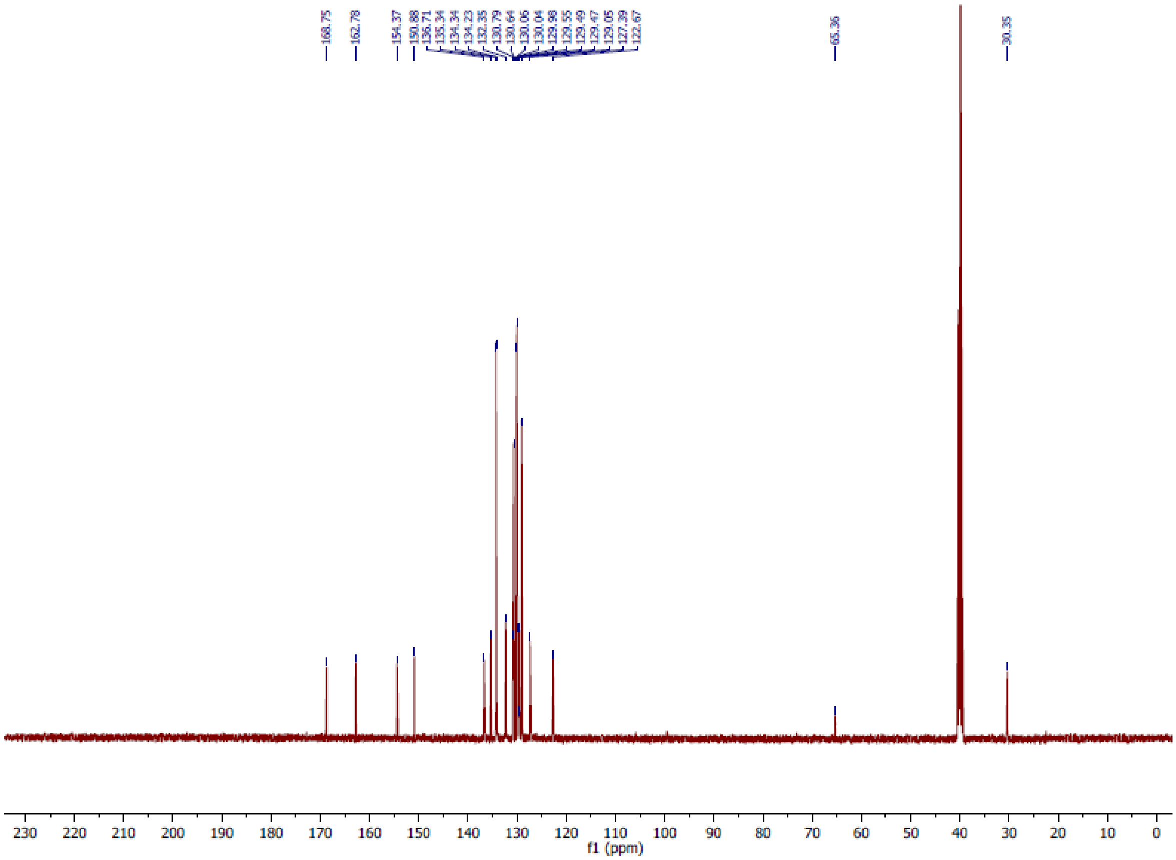

Figure S11. ^{13}C NMR spectrum of complex **1** (DMSO-d_6).

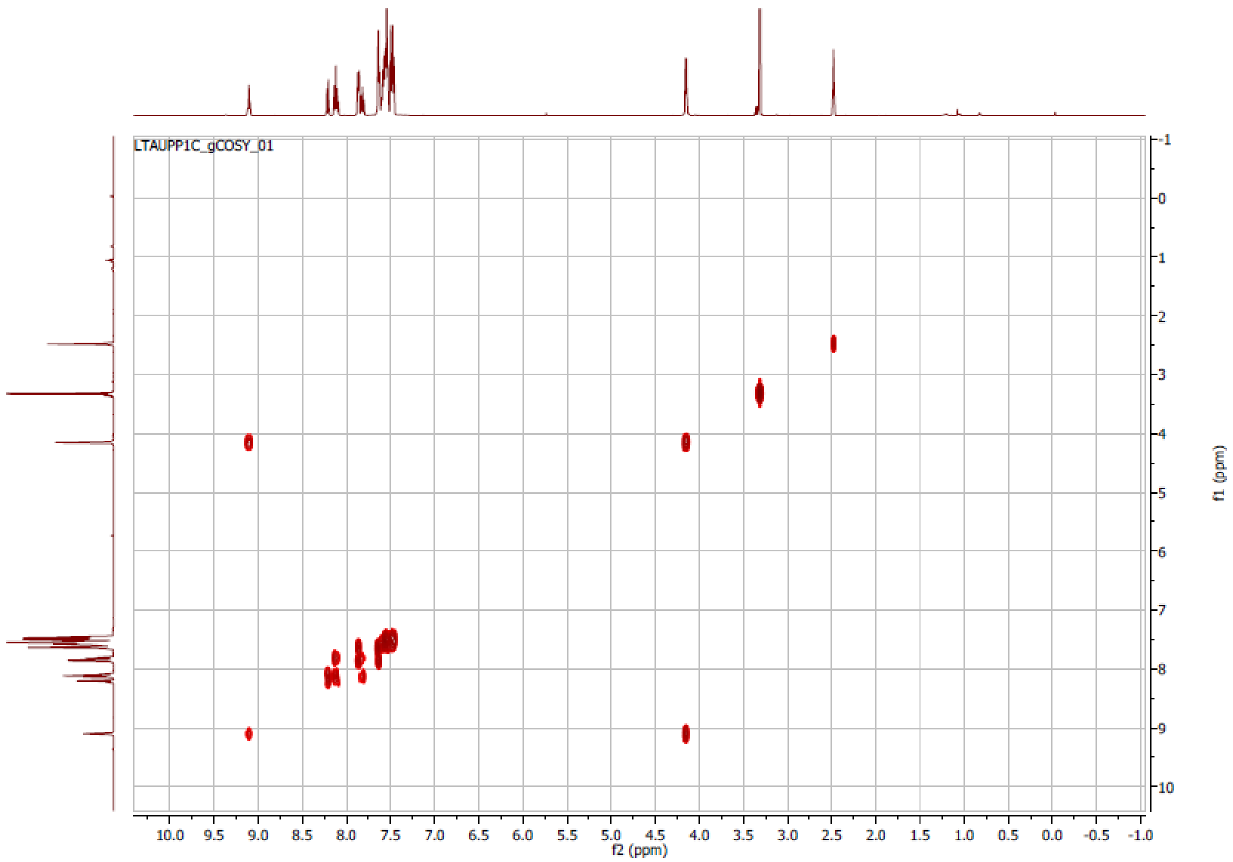

Figure S12. COSY NMR spectrum of complex **1** (DMSO-d_6).

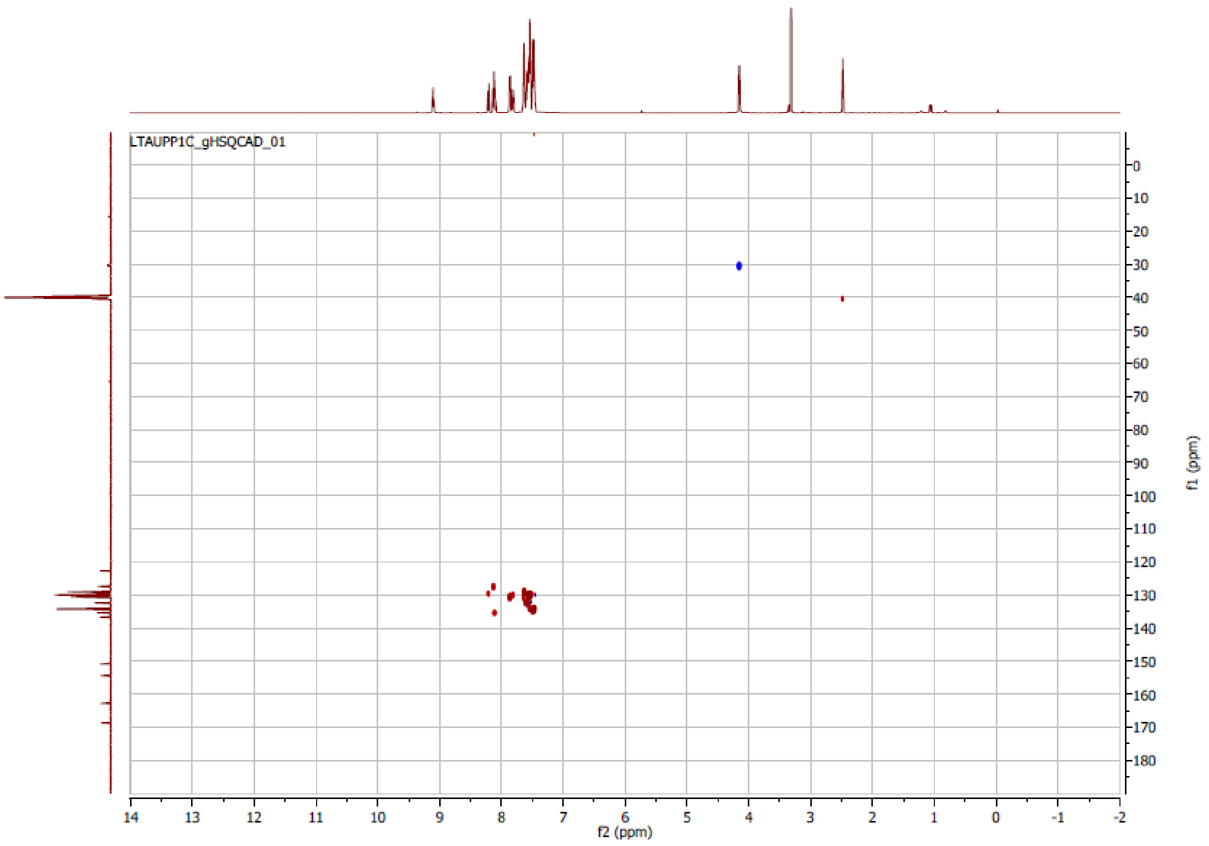

Figure S13. HSQCAD NMR spectrum of complex **1** (DMSO-d_6).

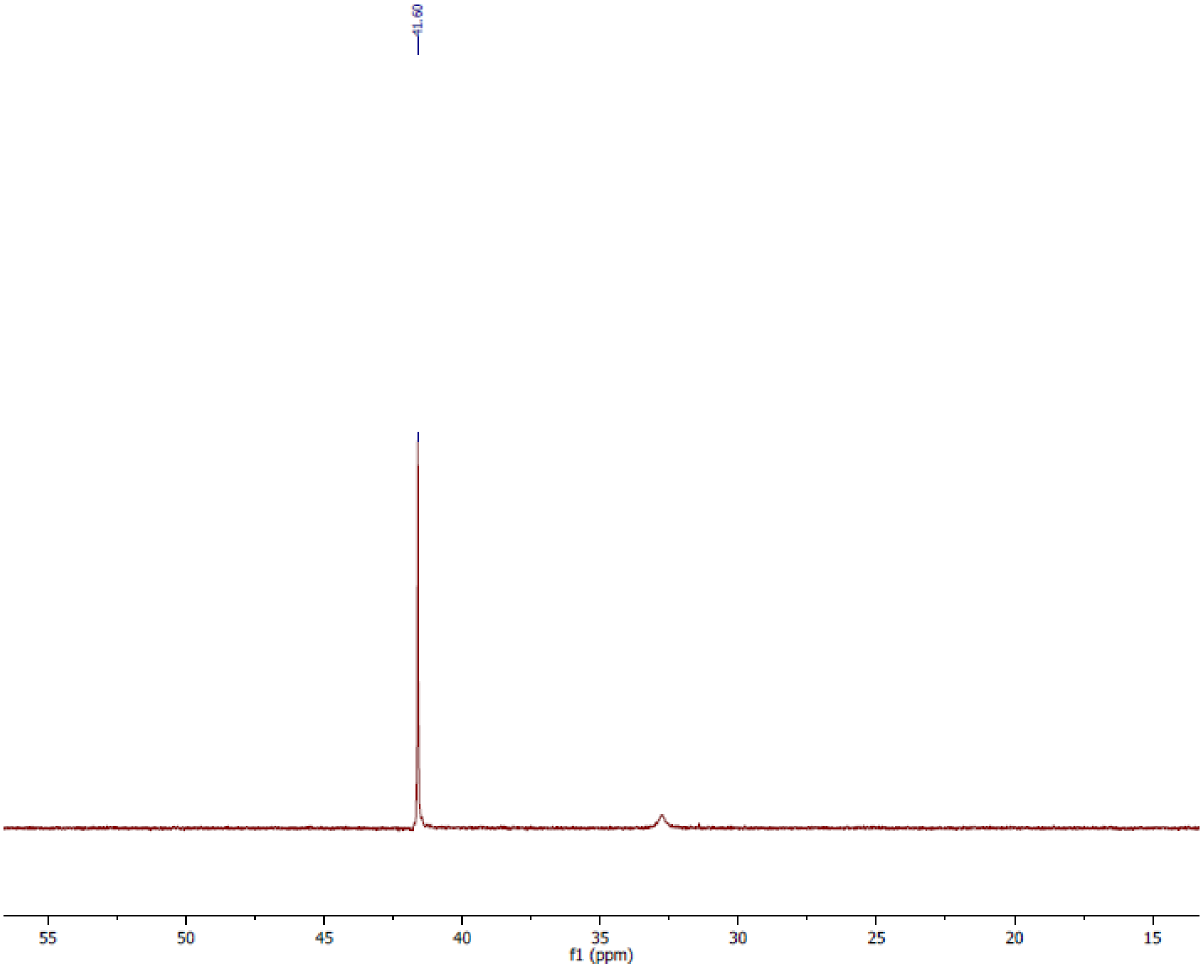

Figure S14. $^{31}P\{1H\}$-NMR spectrum of complex **1** (DMSO-d_6).

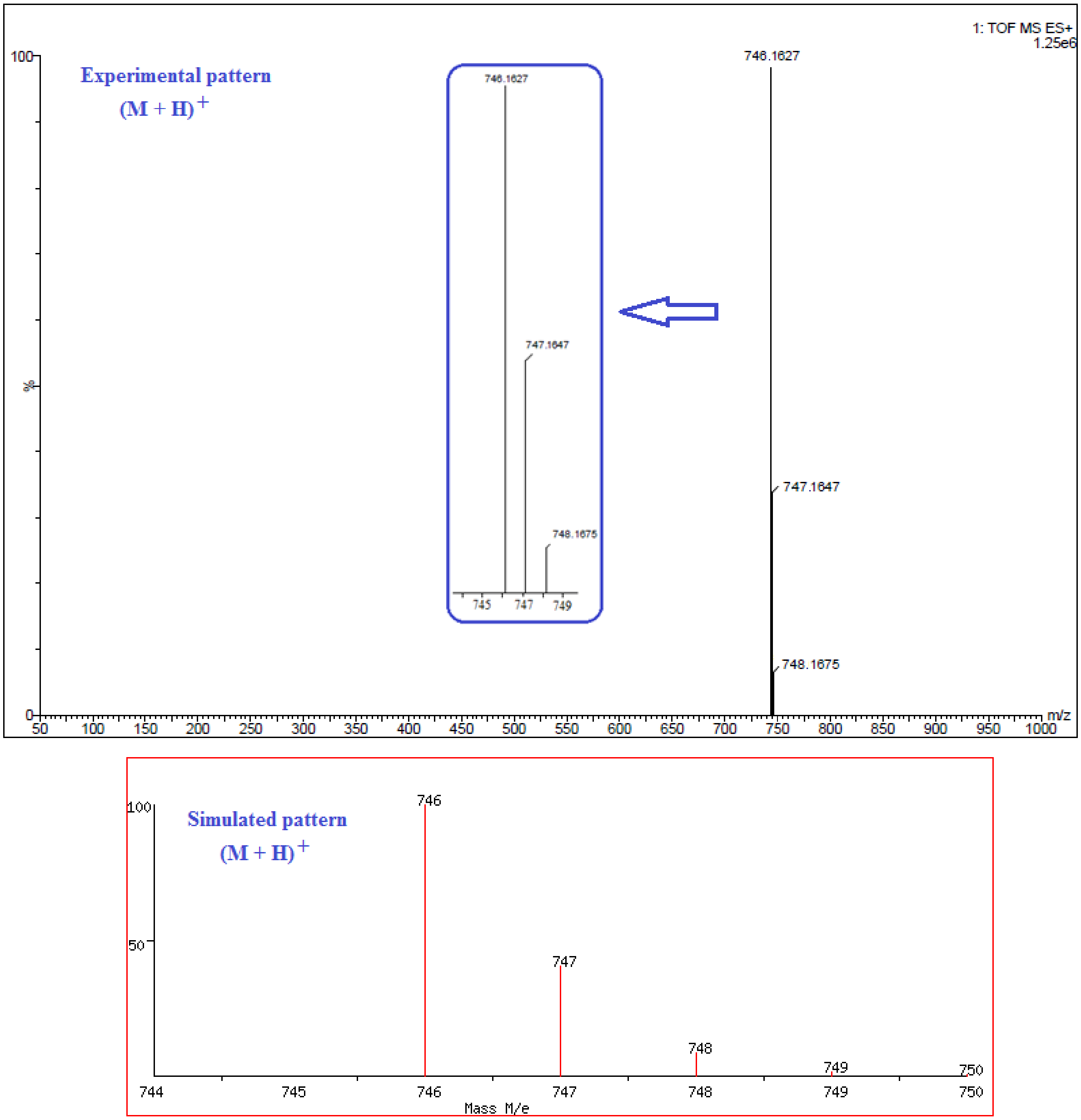

Figure S15. ESI-MS spectrum of complex **1**.

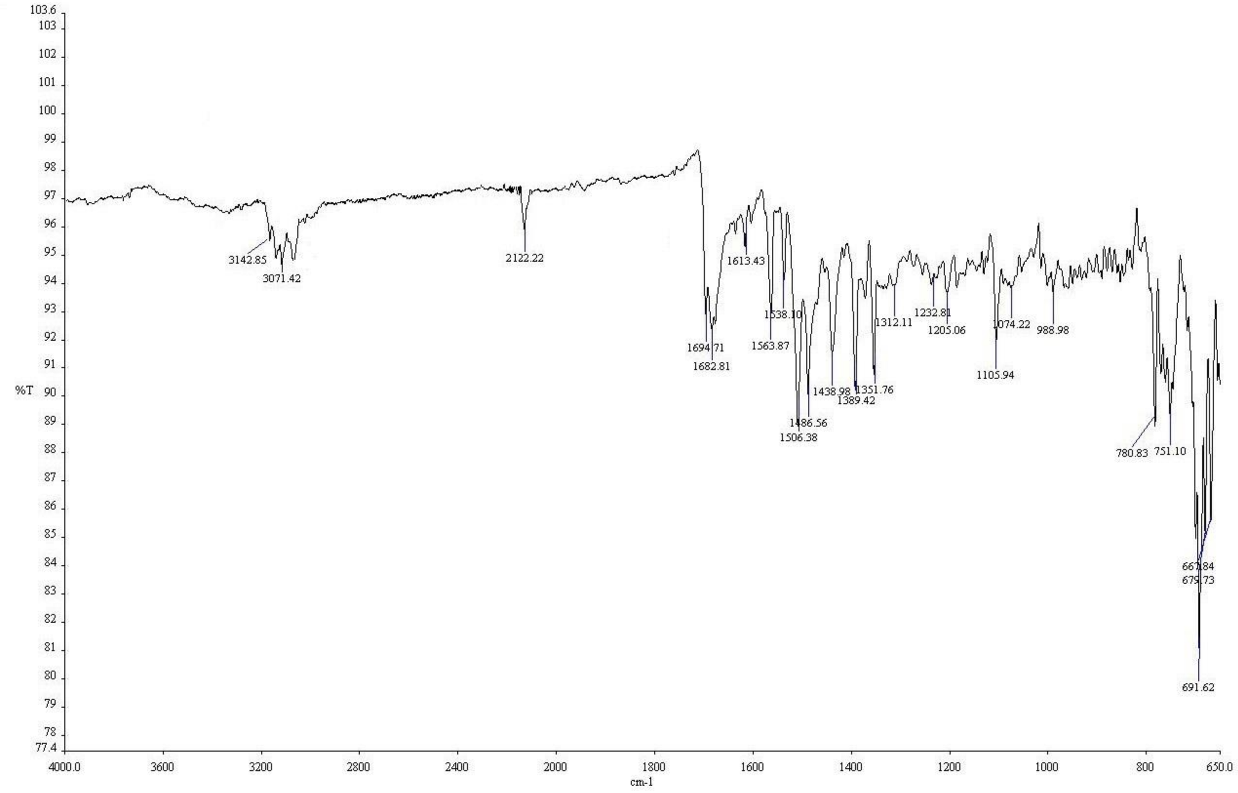

Figure S16. IR spectrum of complex **1**.

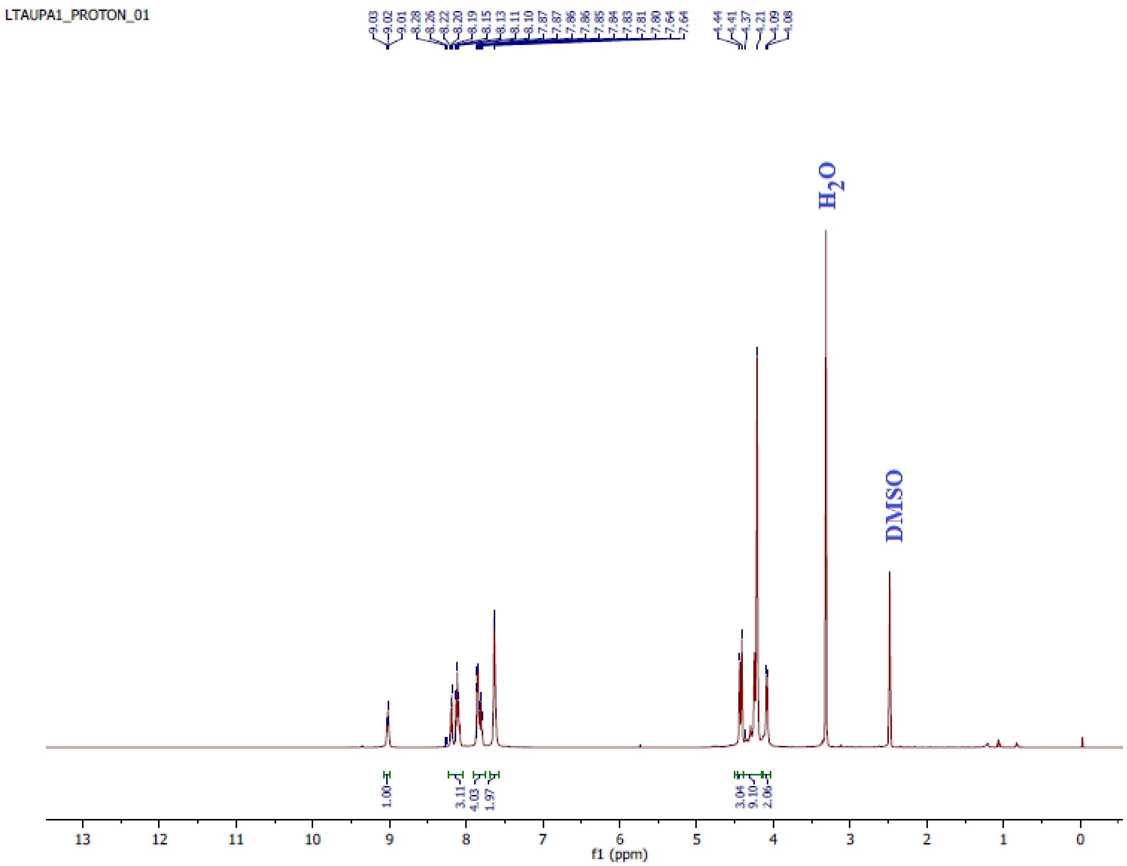

Figure S17. ^{1}H NMR spectrum of complex **2** (DMSO-d_6).

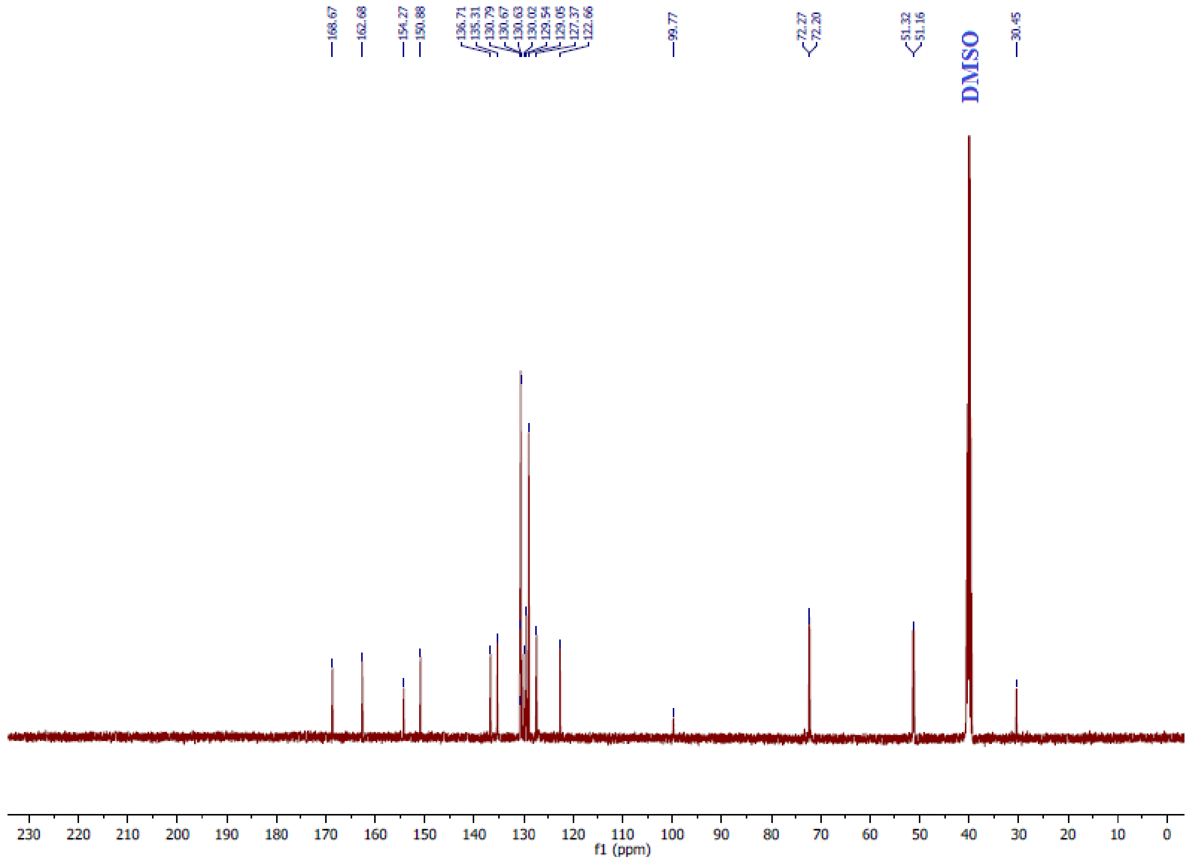

Figure S18. ^{13}C NMR spectrum of complex **2** (DMSO-d_6).

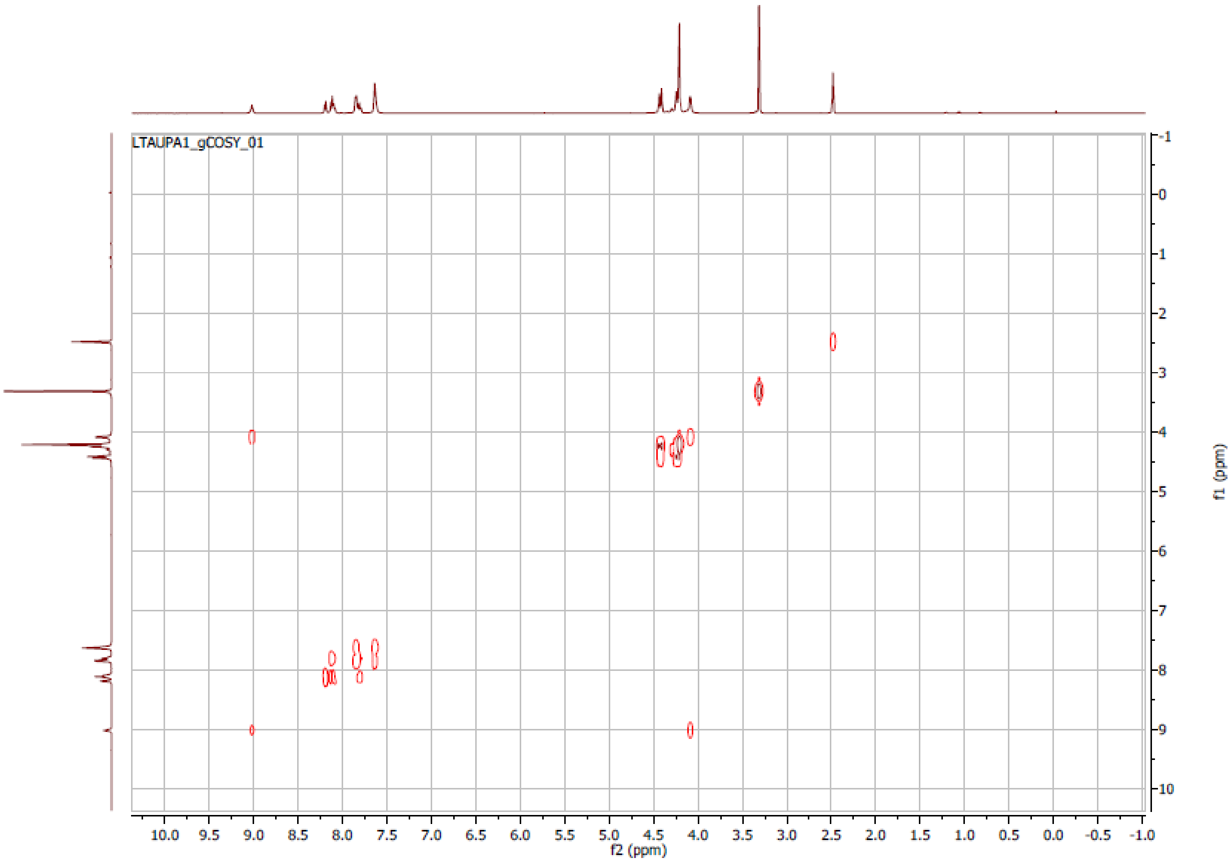

Figure S19. COSY NMR spectrum of complex **2** (DMSO-d_6).

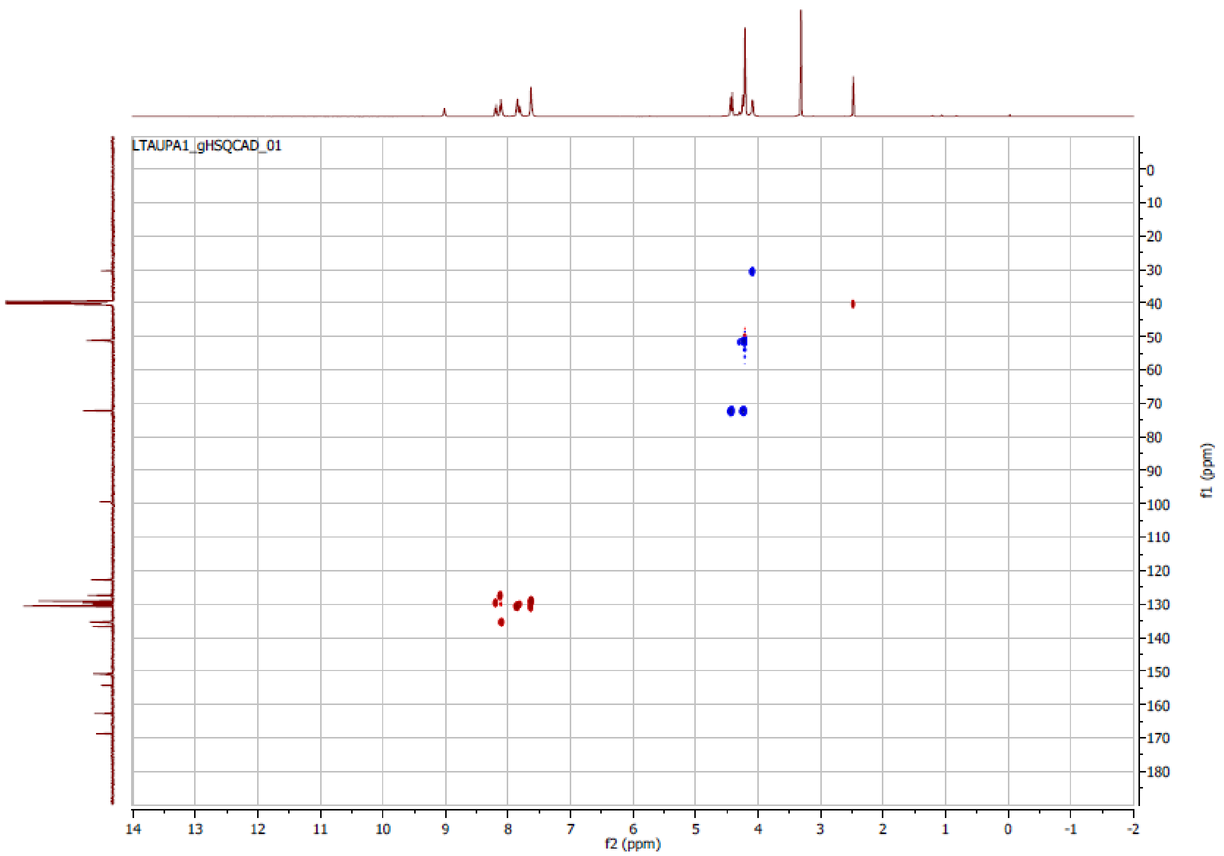

Figure S20. HSQCAD NMR spectrum of complex **2** (DMSO-d_6).

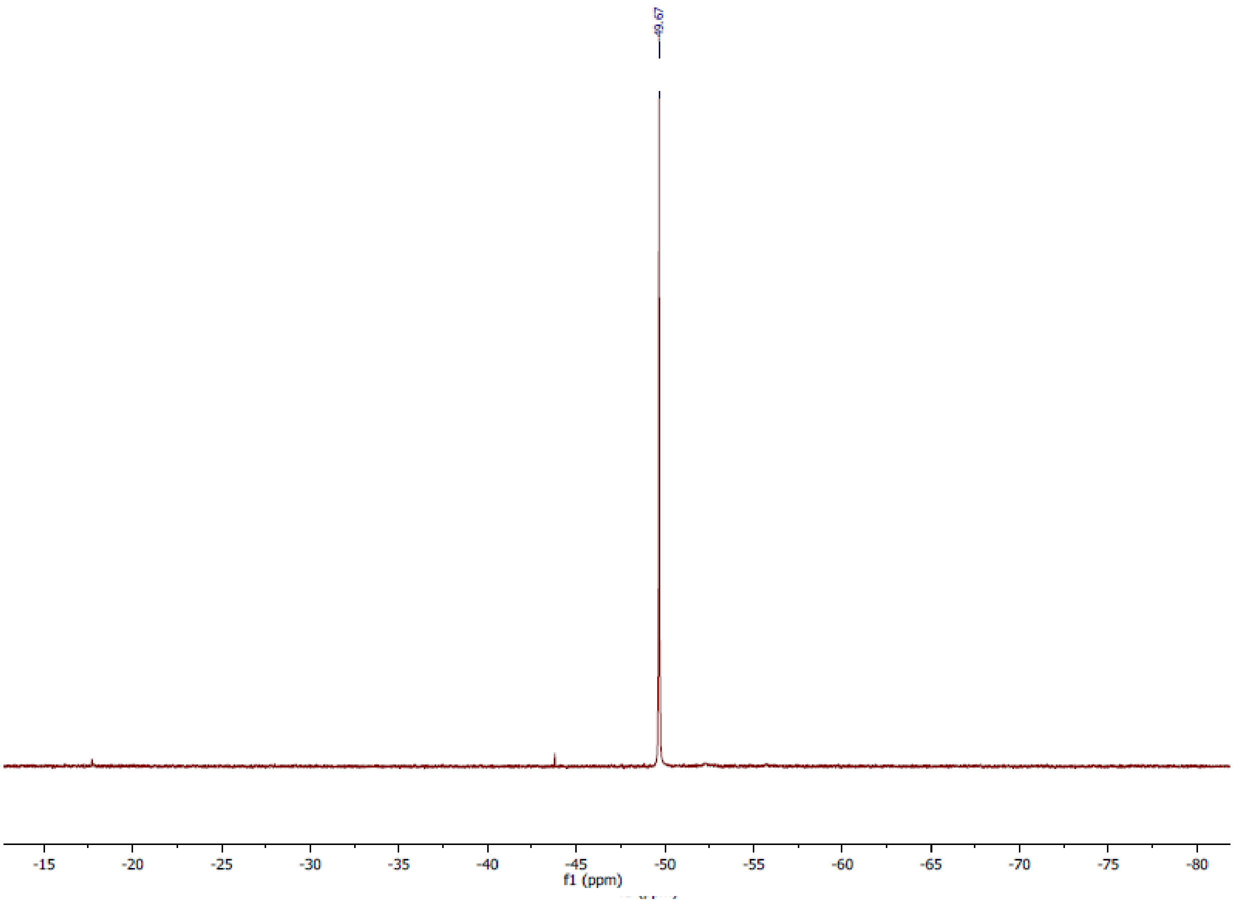

Figure S21. $^{31}P\{1H\}$-NMR spectrum of complex **2** (DMSO-d_6).

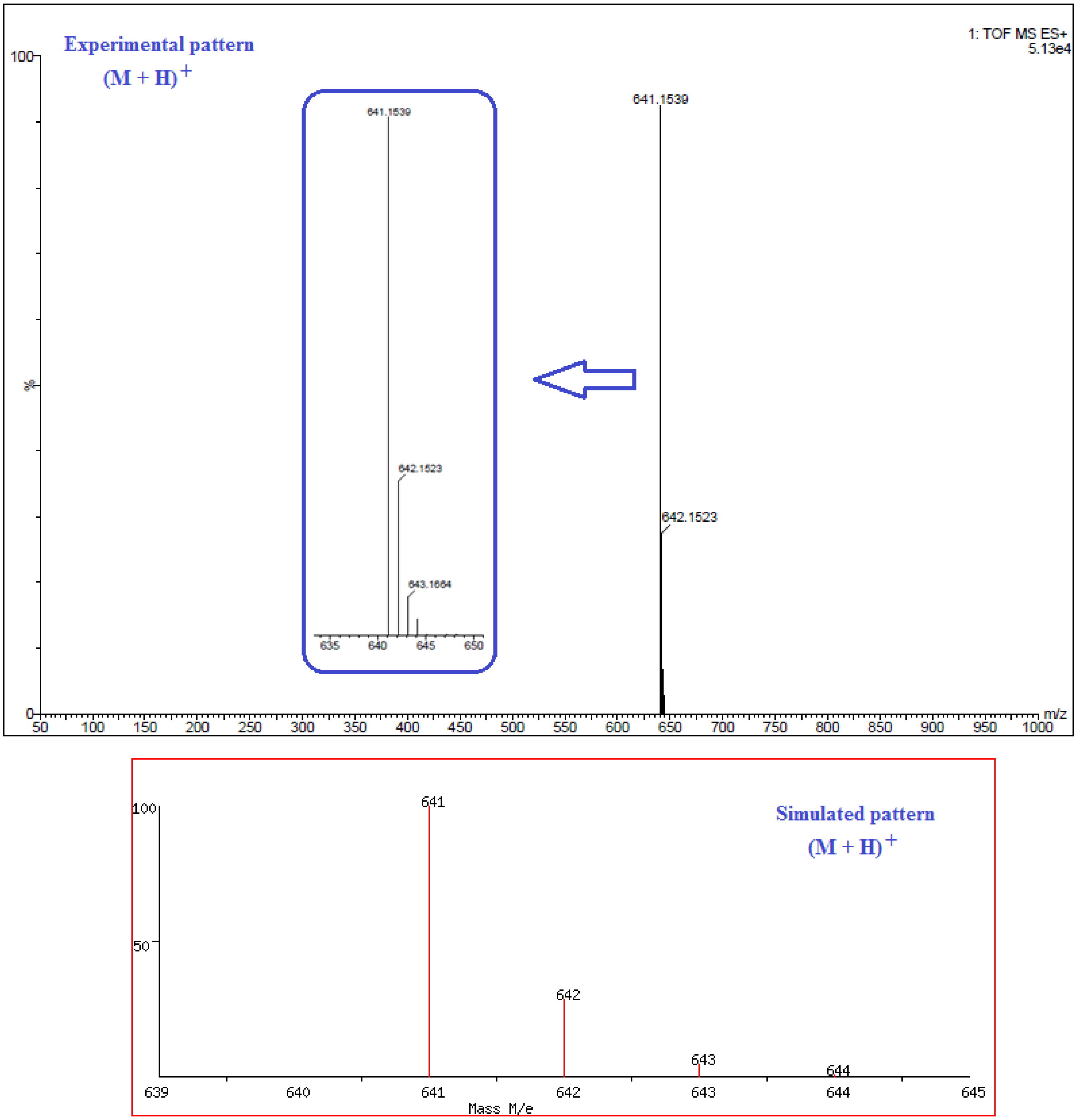

Figure S22. ESI-MS spectrum of complex **2**.

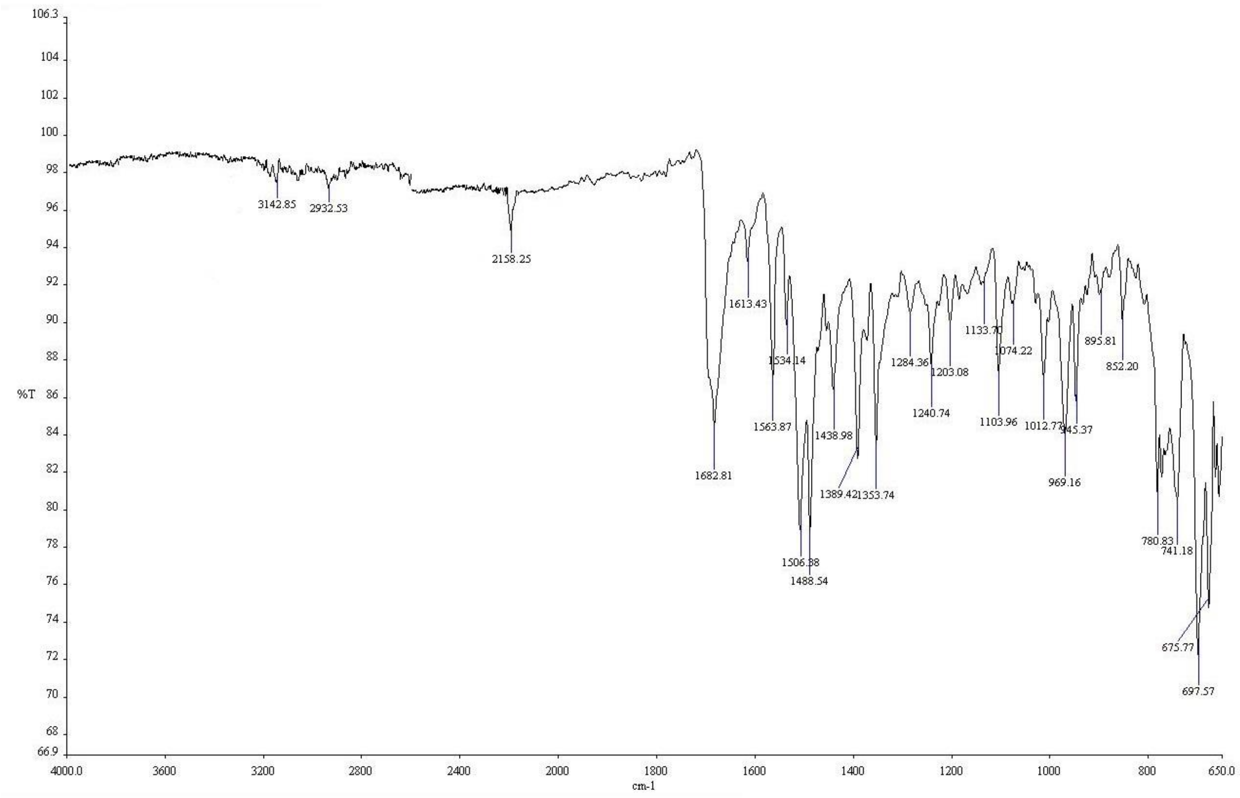

Figure S23. IR spectrum of complex **2**.

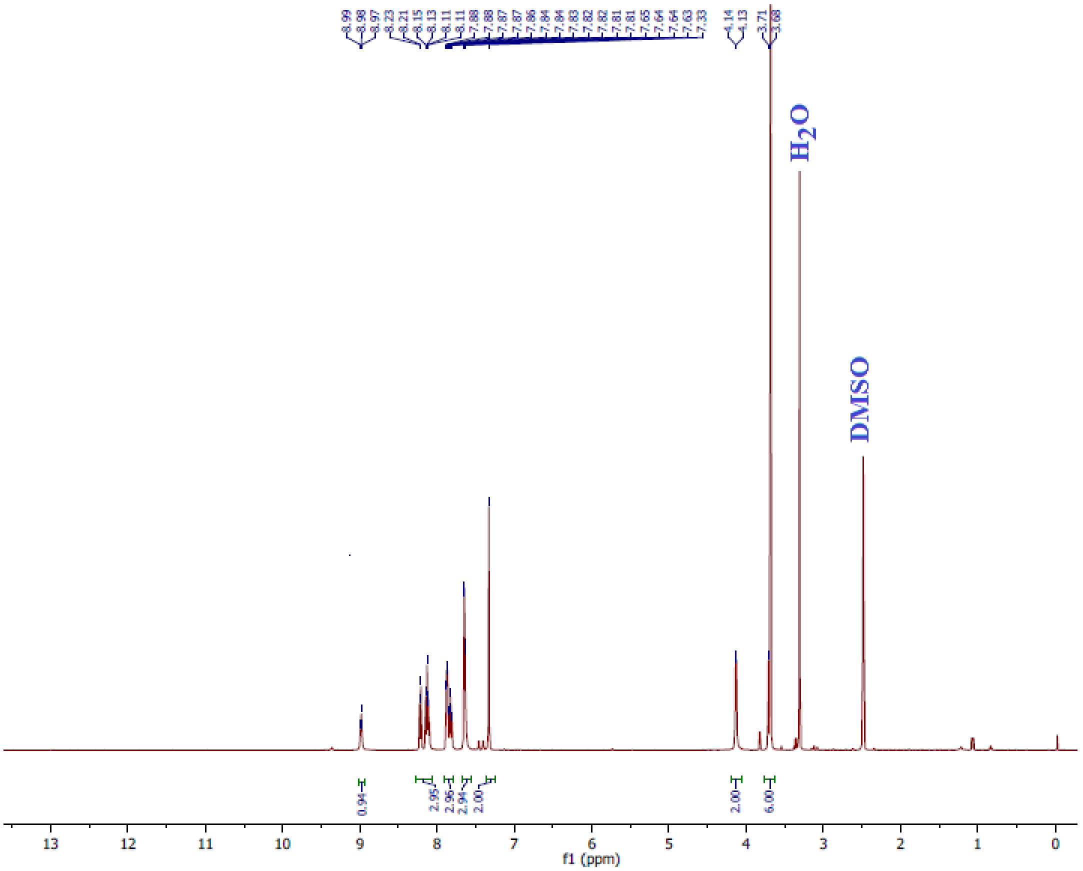

Figure S24. ^{1}H NMR spectrum of complex **3** (DMSO-d_6).

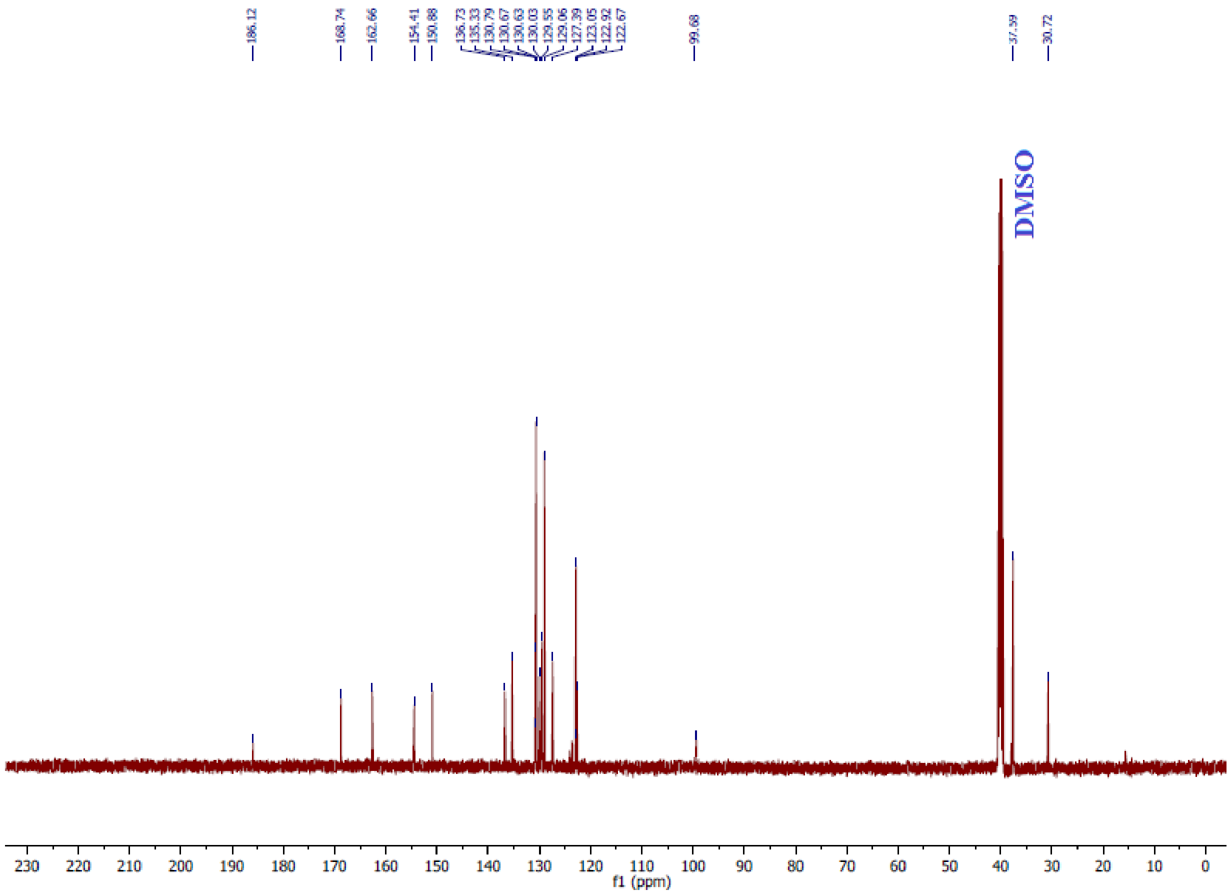

Figure S25. ^{13}C NMR spectrum of complex **3** (DMSO-d_6).

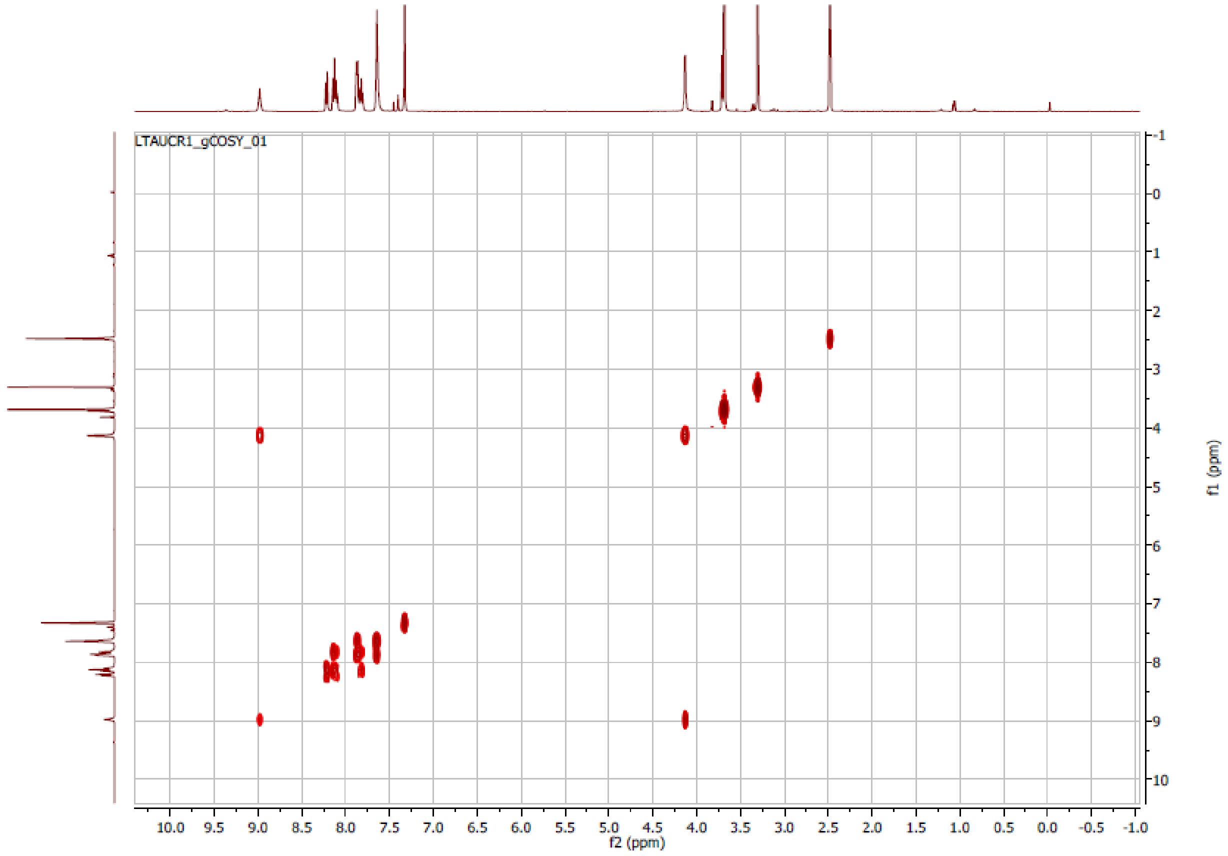

Figure S26. COSY NMR spectrum of complex **3** (DMSO-d_6).

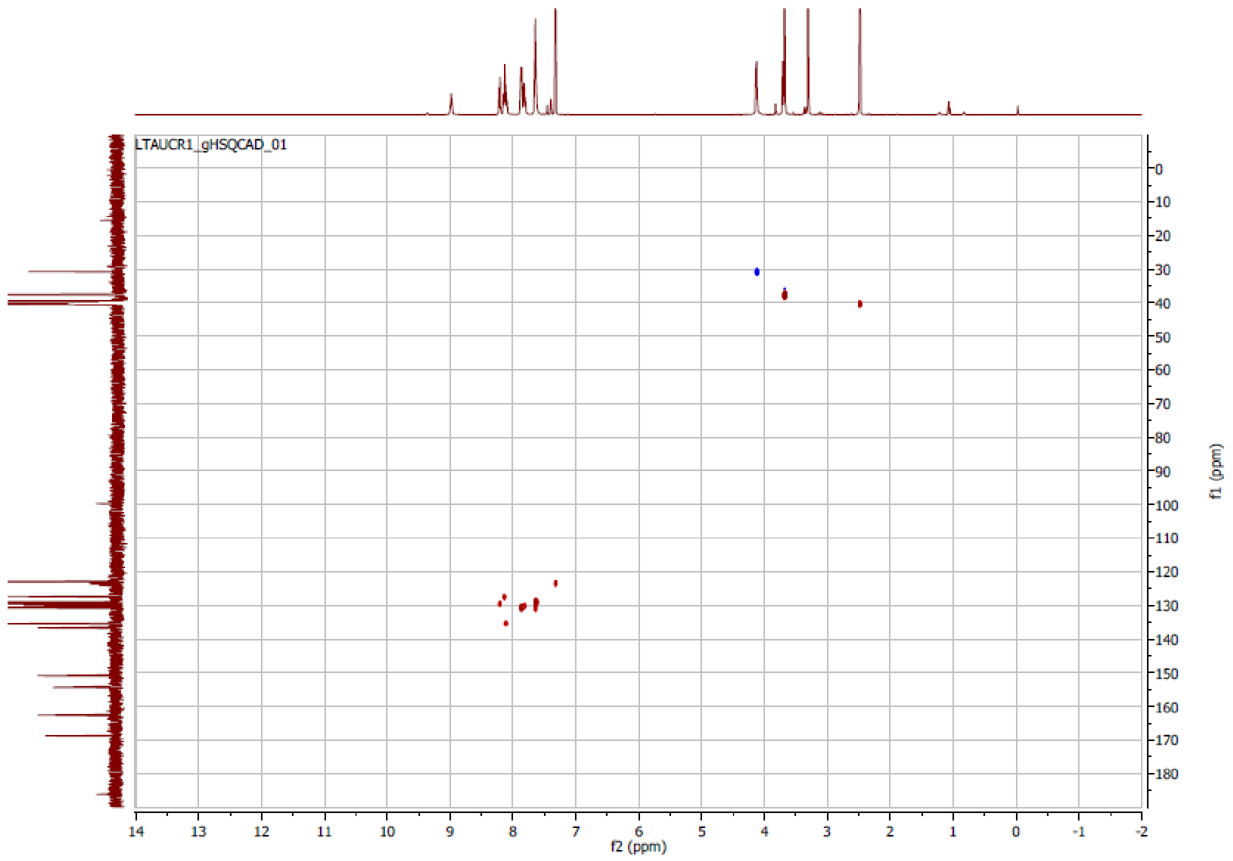

Figure S27. HSQCAD NMR spectrum of complex **3** (DMSO-d_6).

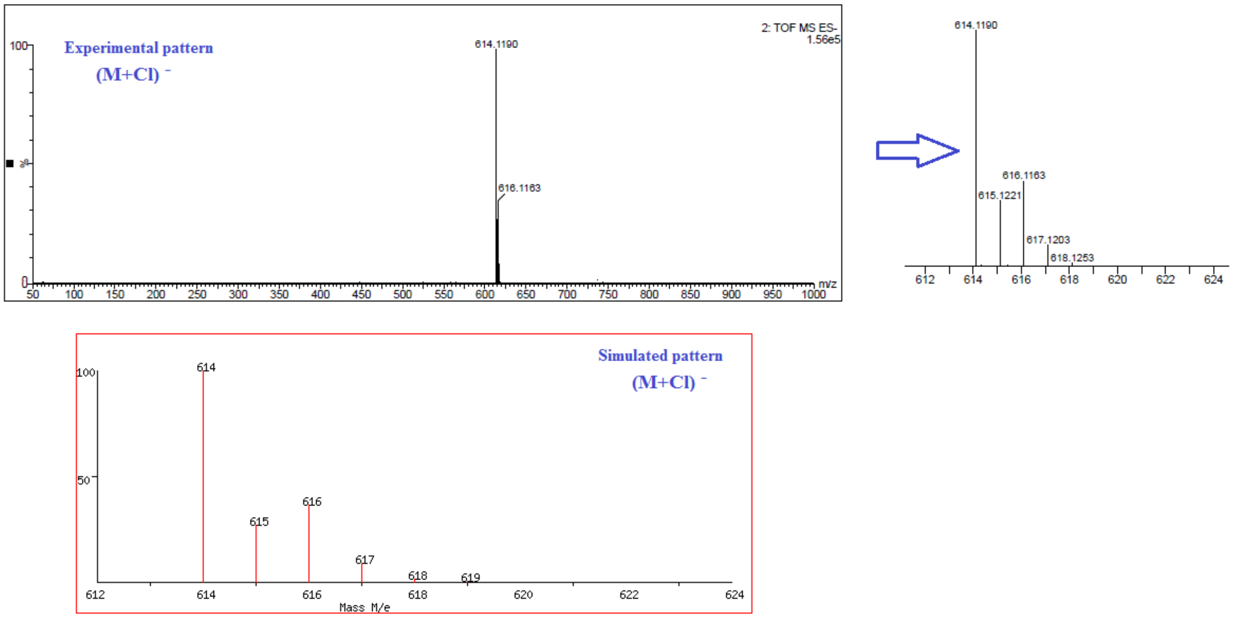

Figure S28. ESI-MS spectrum of complex **3**.

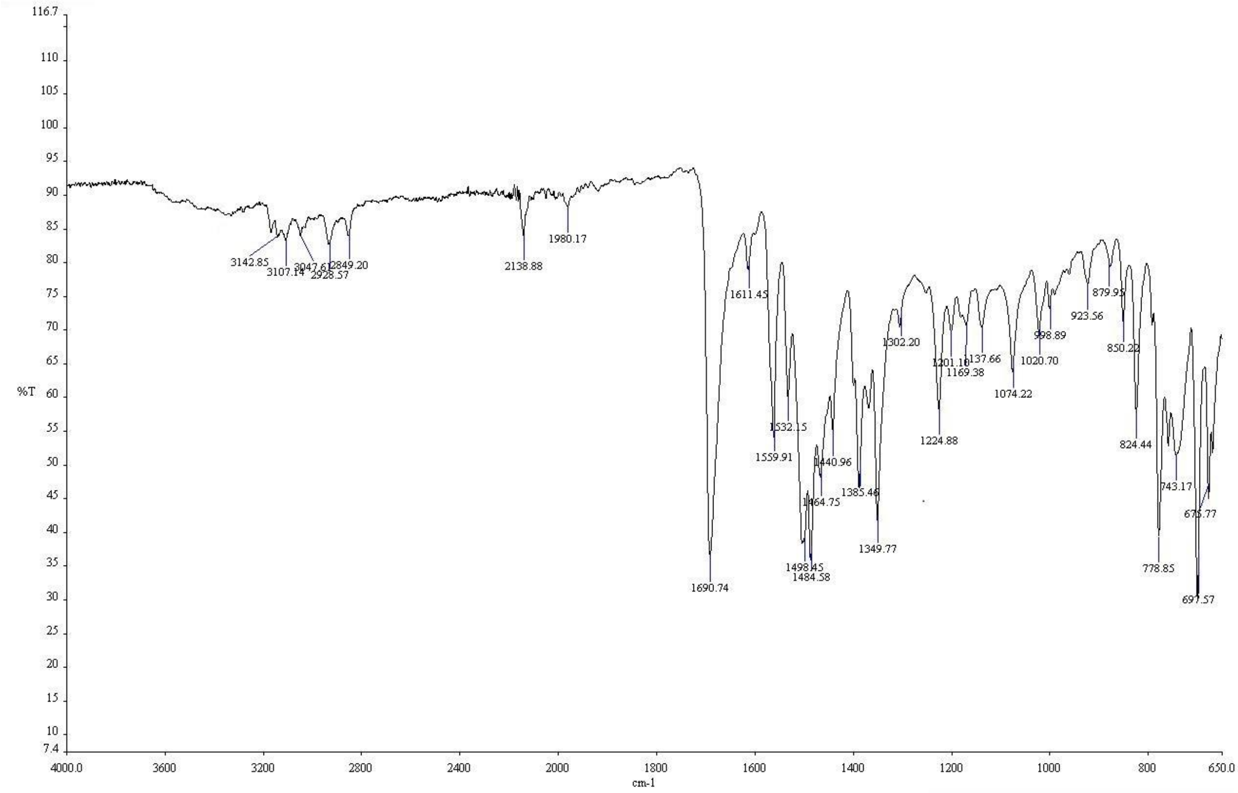

Figure S29. IR spectrum of complex **3**.

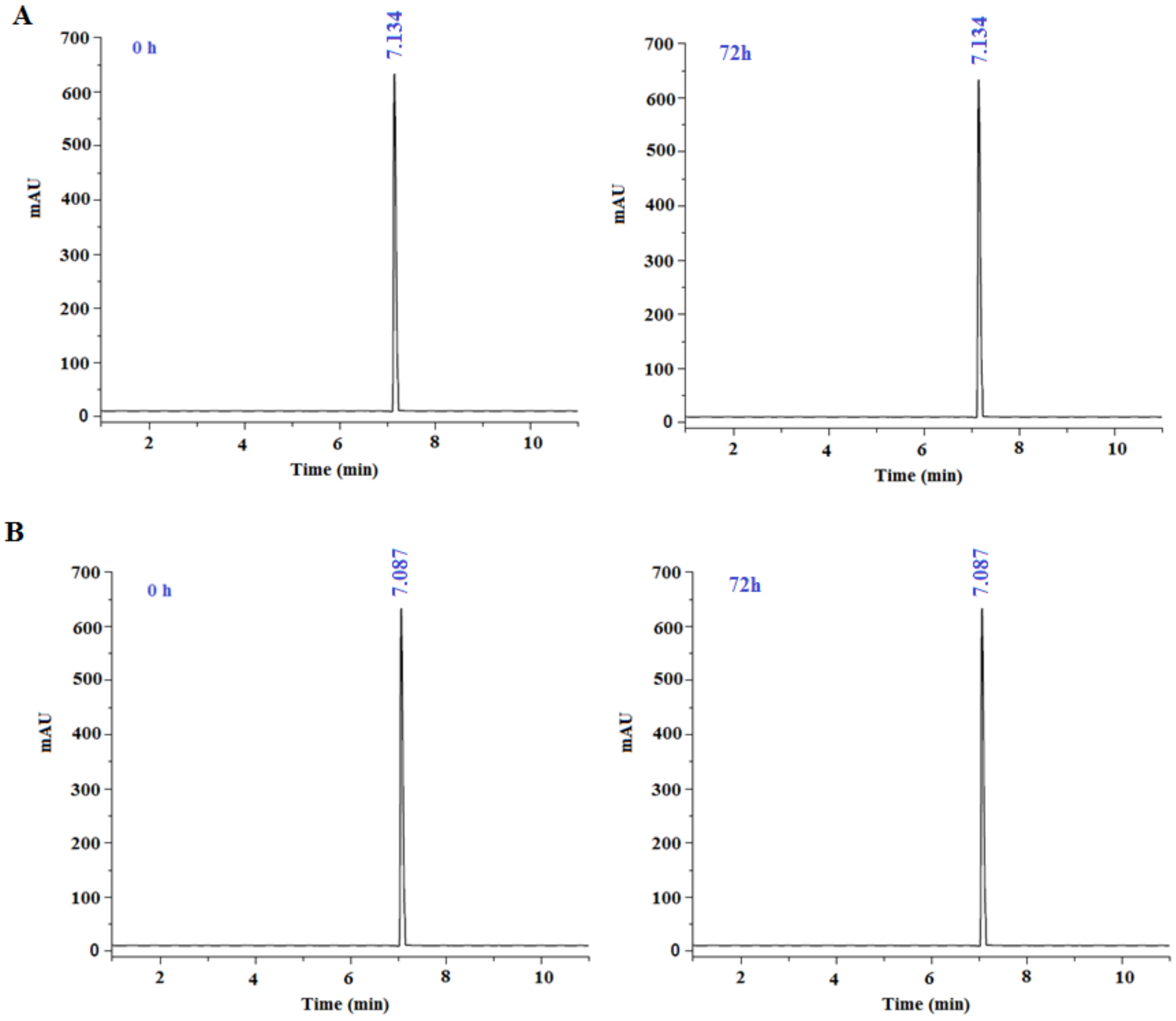

Figure S30. HPLC chromatograms of **L2** in (A) freshly prepared PBS/1% DMSO solution and (B) DMEM (Dulbecco's Modified Eagle's Medium - high glucose)/1% DMSO recorded after 0 h and 72 h incubation at 37 ˚C.

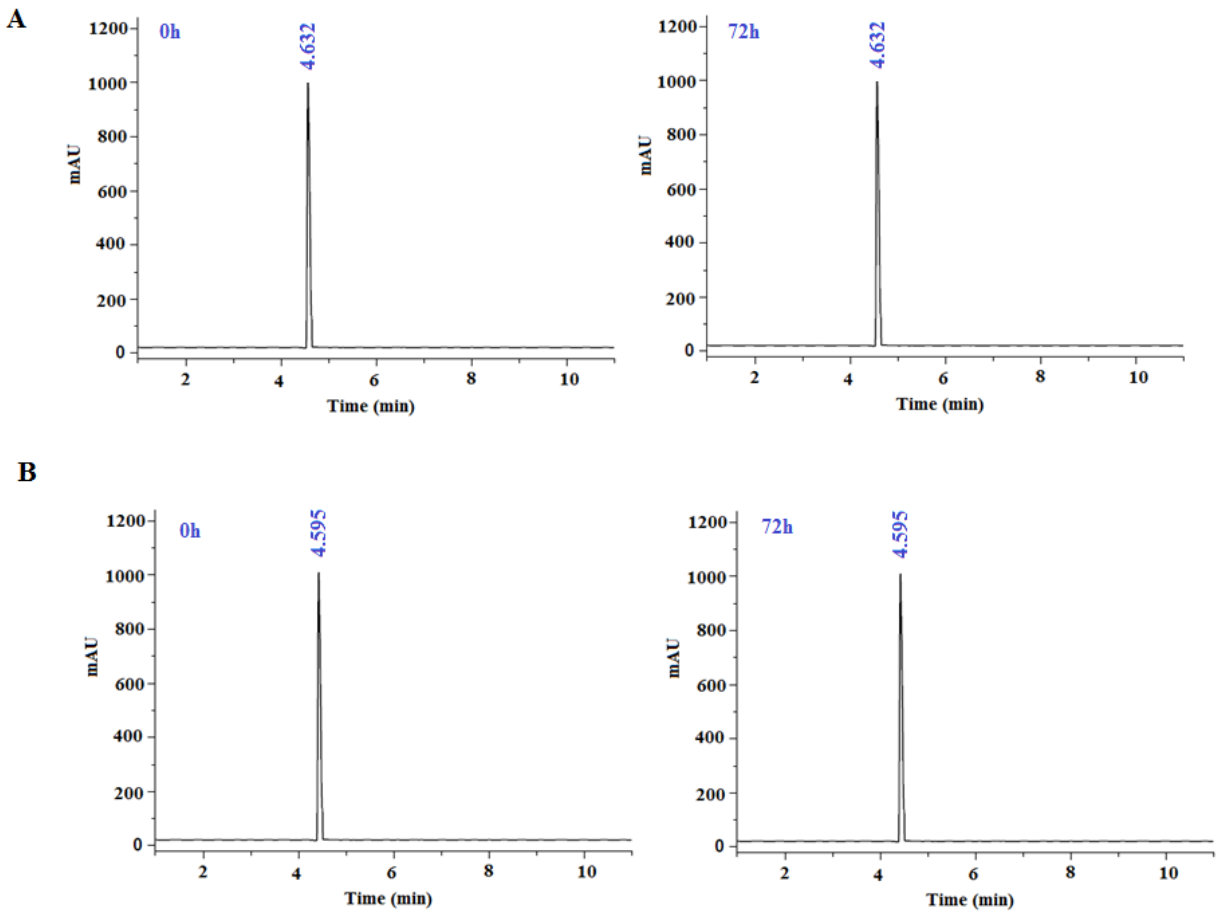

Figure S31. HPLC chromatograms of complex **1** in (A) freshly prepared PBS buffer/1% DMSO solution and (B) DMEM (Dulbecco's Modified Eagle's Medium - high glucose)/1% DMSO recorded after 0 h and 72 h incubation at 37 ˚C.

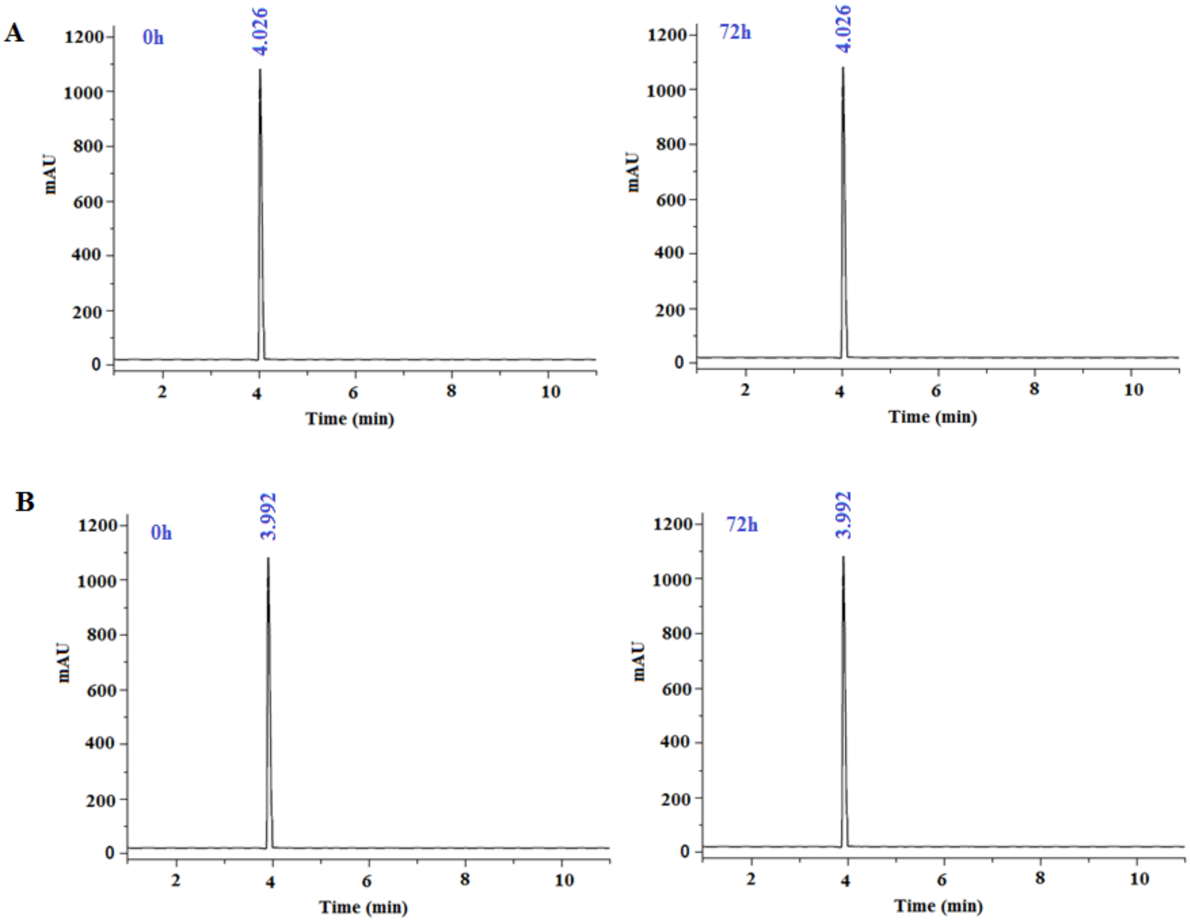

Figure S32. HPLC chromatograms of complex **2** in (A) freshly prepared PBS buffer/1% DMSO solution and (B) DMEM (Dulbecco's Modified Eagle's Medium - high glucose)/1% DMSO recorded after 0 h and 72 h incubation at 37 ˚C.

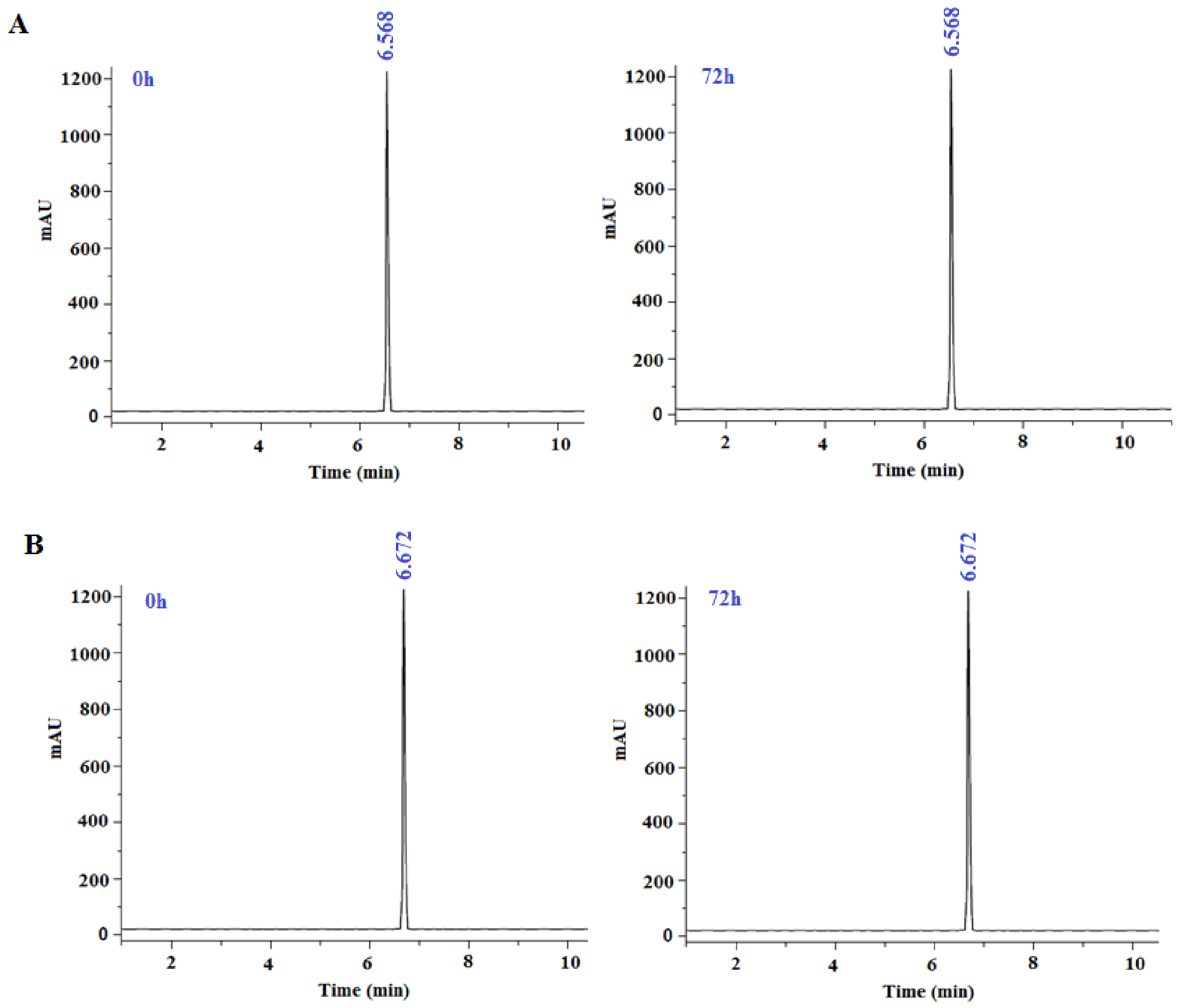

Figure S33. HPLC chromatograms of complex **3** in (A) freshly prepared PBS buffer/1% DMSO solution and (B) DMEM (Dulbecco's Modified Eagle's Medium - high glucose)/1% DMSO recorded after 0 h and 72 h incubation at 37 ˚C.

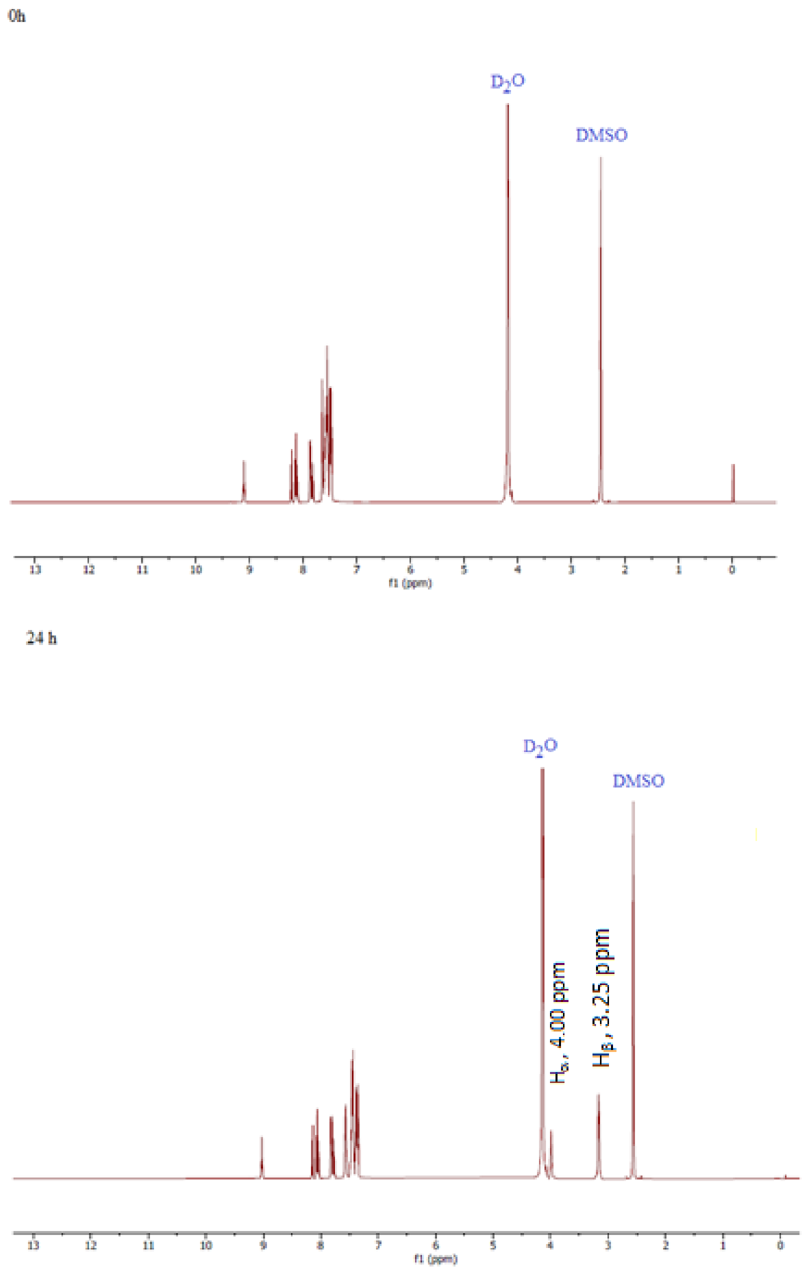

Figure S34. ^{1}H NMR spectrum of complex **1** before (0 h) and after (24 h) addition of one equivalent of L-cysteine in DMSO-d_6: D_2O (1:1).

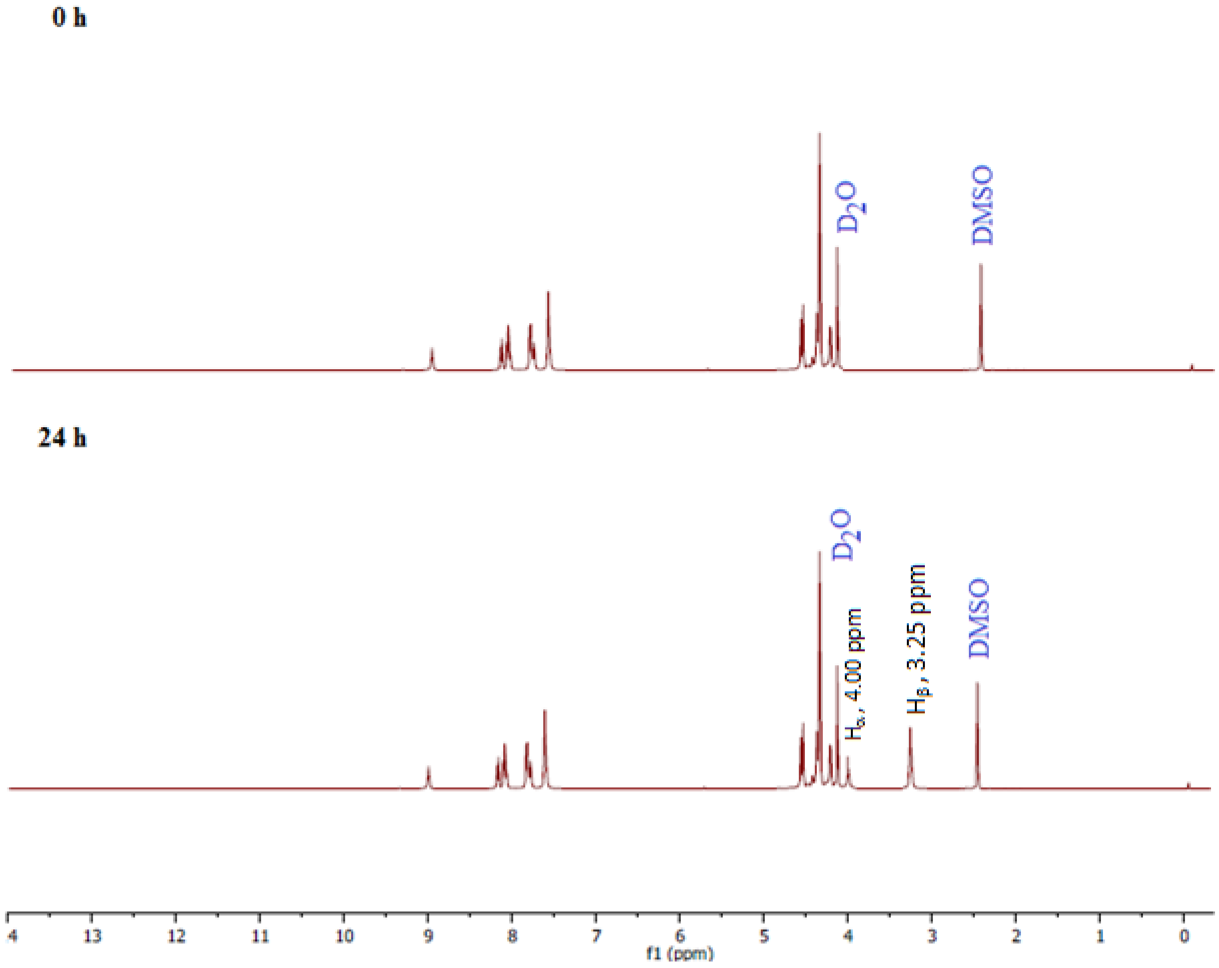

Figure S35. ^{1}H NMR spectrum of complex **2** before (0 h) and after (24 h) addition of one equivalent L-cysteine in DMSO-d_6: D_2O (1:1).

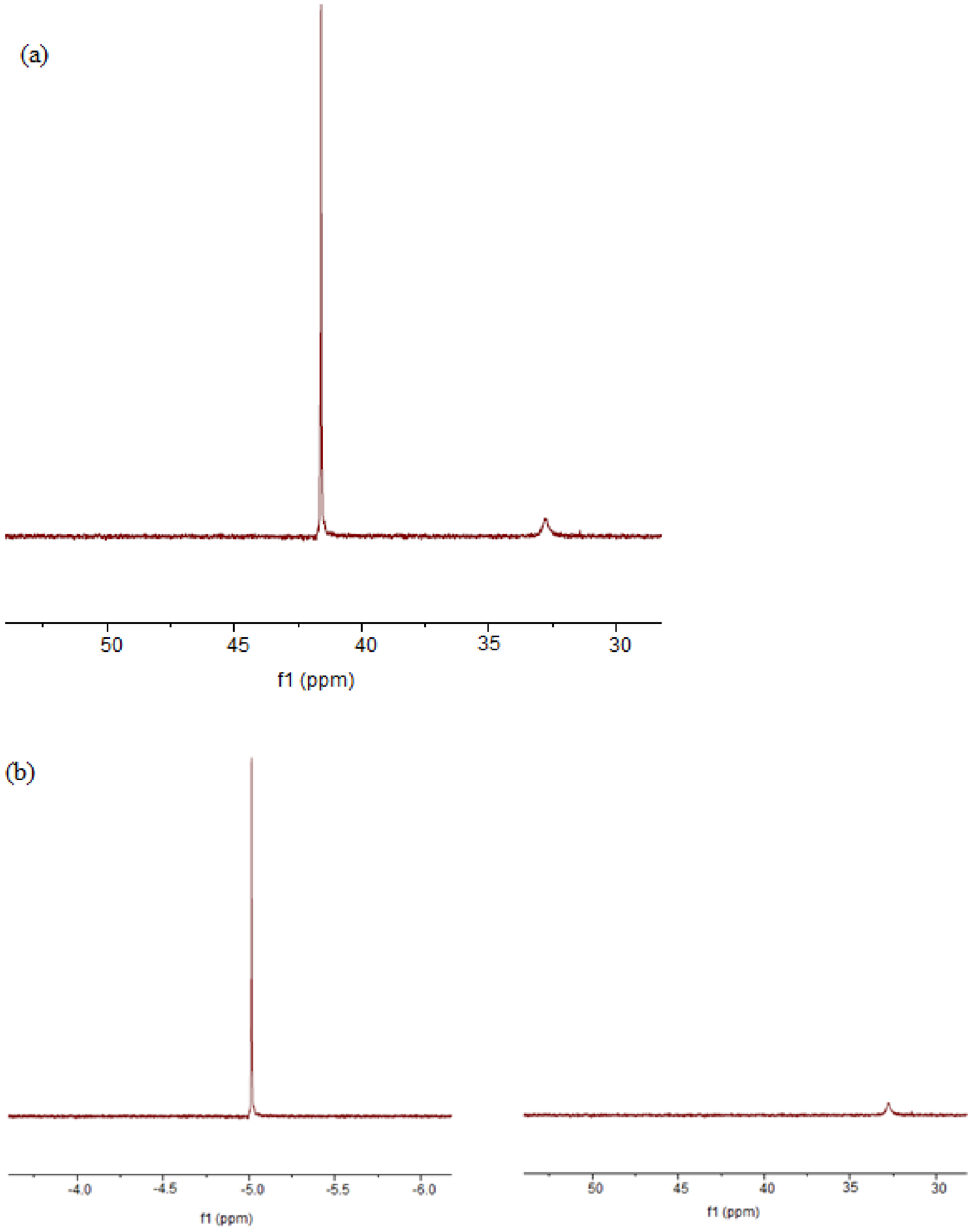

Figure S36. ^{31}P NMR spectrum of complex **1** before (a) and 24 h after (b) the addition of one equivalent of L-cysteine in DMSO-d_6: D_2O (1:1).

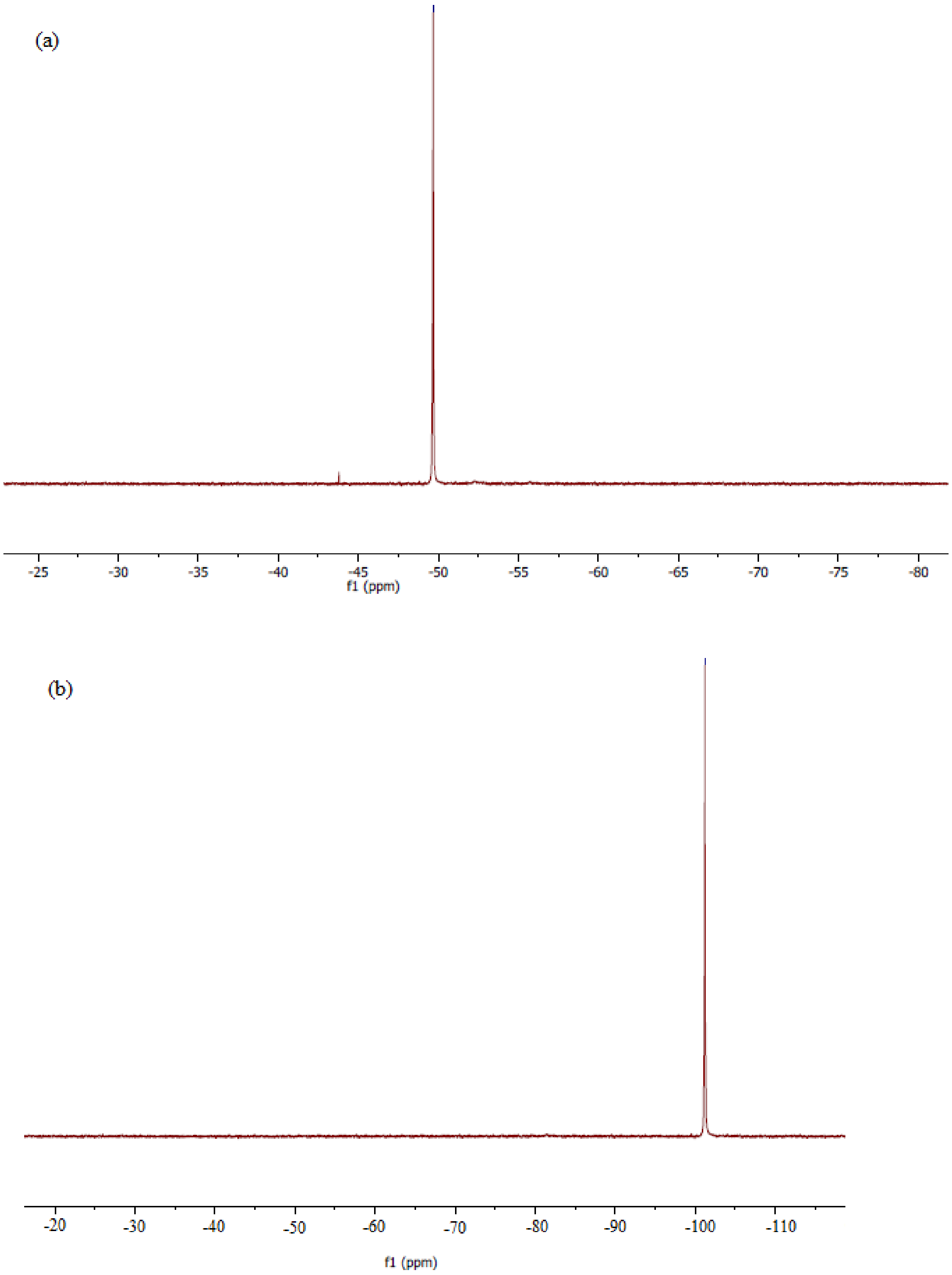

Figure S37. ^{31}P NMR spectrum of complex **2** before (a) and 24 h after (b) the addition of one equivalent of L-cysteine in DMSO-d_6: D_2O (1:1).

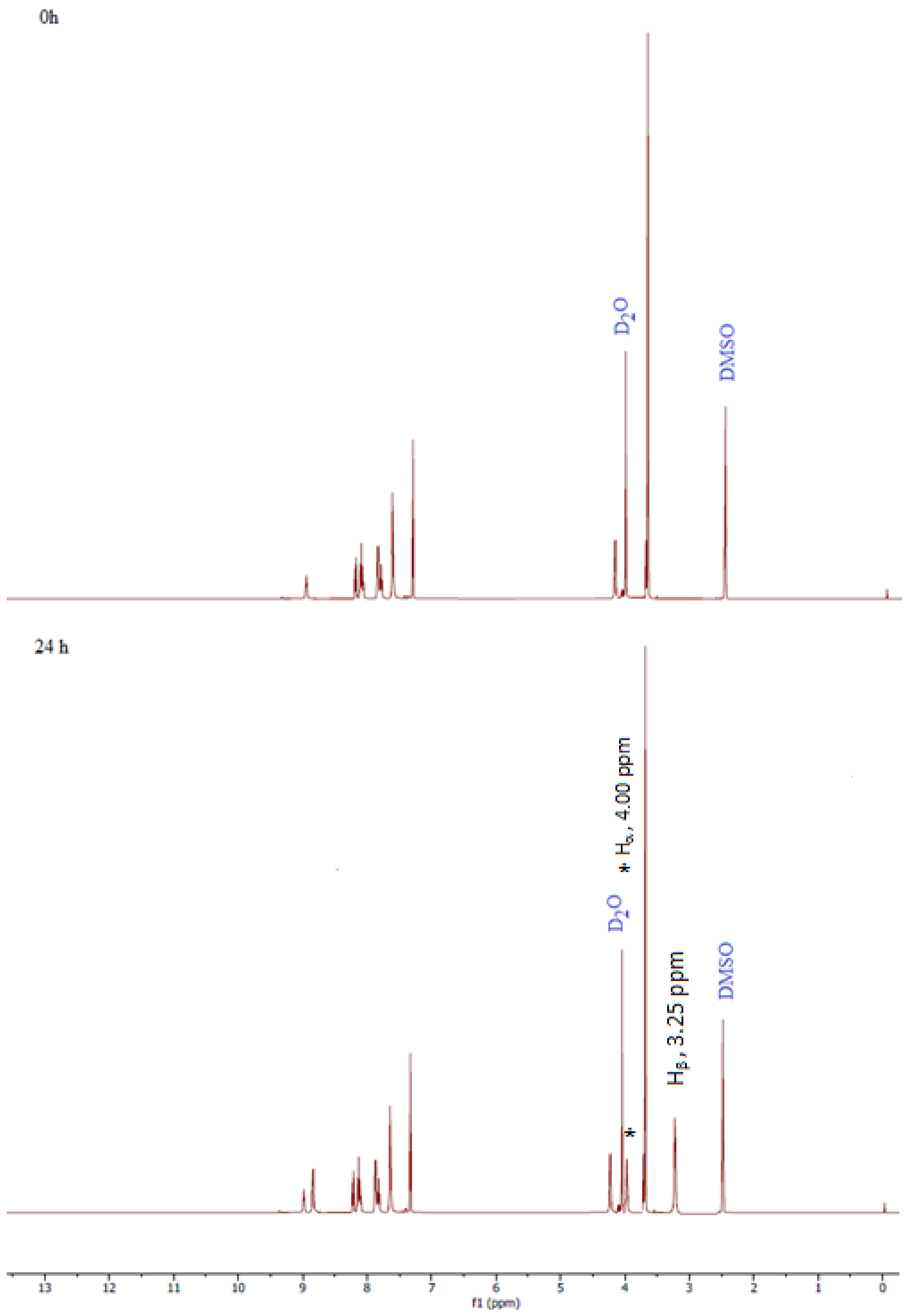

Figure S38. ^{1}H NMR spectrum of complex **3** before (0 h) and after (24 h) addition of one equivalent of L-cysteine in DMSO-d_6: D_2O (1:1).
